# Antioxidative and Metabolic Contribution to Salinity Stress Responses in Two Rapeseed Cultivars during the Early Seedling Stage

**DOI:** 10.3390/antiox10081227

**Published:** 2021-07-30

**Authors:** Ali Mahmoud El-Badri, Maria Batool, Ibrahim A. A. Mohamed, Zongkai Wang, Ahmed Khatab, Ahmed Sherif, Hasan Ahmad, Mohammad Nauman Khan, Hamada Mohamed Hassan, Ibrahim M. Elrewainy, Jie Kuai, Guangsheng Zhou, Bo Wang

**Affiliations:** 1 MOA Key Laboratory of Crop Ecophysiology and Farming System in the Middle Reaches of the Yangtze River, College of Plant Science & Technology, Huazhong Agricultural University, Wuhan 430070, China; alyelbadry@webmail.hzau.edu.cn (A.M.E.-B.); maria.batool@webmail.hzau.edu.cn (M.B.); iaa04@fayoum.edu.eg (I.A.A.M.); wangzongkai@webmail.hzau.edu.cn (Z.W.); ahmedkhatab@webmail.hzau.edu.cn (A.K.); Sherif@webmail.hzau.edu (A.S.); nauman@webmail.hzau.edu.cn (M.N.K.); 105042014160@mail.hzau.edu.cn (J.K.); zhougs@mail.hzau.edu.cn (G.Z.); 2 Field Crops Research Institute, Agricultural Research Center (ARC), Giza 12619, Egypt; hhassan997@yahoo.com (H.M.H.); imelrewainy@gmail.com (I.M.E.); 3 Botany Department, Faculty of Agriculture, Fayoum University, Fayoum 63514, Egypt; 4 National Gene Bank, Agricultural Research Center (ARC), Giza 12619, Egypt; hasanngb@webmail.hzau.edu.cn

**Keywords:** *Brassica napus*, salinity stress, antioxidant enzymes, osmolytes, ROS, metabolites

## Abstract

Measuring metabolite patterns and antioxidant ability is vital to understanding the physiological and molecular responses of plants under salinity. A morphological analysis of five rapeseed cultivars showed that Yangyou 9 and Zhongshuang 11 were the most salt-tolerant and -sensitive, respectively. In Yangyou 9, the reactive oxygen species (ROS) level and malondialdehyde (MDA) content were minimized by the activation of antioxidant enzymes such as superoxide dismutase (SOD), peroxidase (POD), catalase (CAT), and ascorbate peroxidase (APX) for scavenging of over-accumulated ROS under salinity stress. Furthermore, Yangyou 9 showed a significantly higher positive correlation with photosynthetic pigments, osmolyte accumulation, and an adjusted Na^+^/K^+^ ratio to improve salt tolerance compared to Zhongshuang 11. Out of 332 compounds identified in the metabolic profile, 225 metabolites were filtrated according to *p* < 0.05, and 47 metabolites responded to salt stress within tolerant and sensitive cultivars during the studied time, whereas 16 and 9 metabolic compounds accumulated during 12 and 24 h, respectively, in Yangyou 9 after being sown in salt treatment, including fatty acids, amino acids, and flavonoids. These metabolites are relevant to metabolic pathways (amino acid, sucrose, flavonoid metabolism, and tricarboxylic acid cycle (TCA), which accumulated as a response to salinity stress. Thus, Yangyou 9, as a tolerant cultivar, showed improved antioxidant enzyme activity and higher metabolite accumulation, which enhances its tolerance against salinity. This work aids in elucidating the essential cellular metabolic changes in response to salt stress in rapeseed cultivars during seed germination. Meanwhile, the identified metabolites can act as biomarkers to characterize plant performance in breeding programs under salt stress. This comprehensive study of the metabolomics and antioxidant activities of *Brassica napus* L. during the early seedling stage is of great reference value for plant breeders to develop salt-tolerant rapeseed cultivars.

## 1. Introduction

For thousands of years, rapeseed (*Brassica species*) has been planted for its high production of edible oils and its economic and significant nutritional value [1]. Canada is the largest rapeseed producer, followed by China and India [2]. Different *Brassica* species are grown or adapted to different climates; in particular, brassica crops are extensively cultivated in arid and semi-arid regions, where the accumulation of salts negatively affects germination, early seedling growth, and productivity [3].

Climate changes such as drought, salinity, and temperature are a threat to food production by limiting crop productivity [4]. Salinity is one of the main abiotic stresses that negatively affects agricultural crop productivity, by impairing germination, plant vigor, and crop yield [5]. All over the world, salinity causes damage to more than 20% of cultivated land in addition to 33% of irrigated agricultural land. Every year, about 1.5 million hectares are not cultivated due to high salinity levels in the soil. There are many reasons for increased salinity, incloding low precipitation, high surface evaporation, weathering of native rocks, and poor cultural practices [6]. It is expected that, by 2050, more than 50% of agricultural land could be damaged by salinity [7]. During the plant life cycle, seed germination and seedling vigor are complicated and critical phenomena that are highly affected by various environmental stresses, especially salinity [8]. Osmotic stress and ion toxicity are among the main reasons behind the restriction of plant growth in salinized soils, due to the higher levels of salt in the soil, which restricts plants from extracting water from the soil and inside the plants themselves, which then causes nutritional imbalance and oxidative stress [9,10]. Additionally, Na^+^ can replace ions, particularly K^+^, in key enzymatic reactions, which affects cytosol and organelle metabolism due to the Na^+^/K^+^ ratio, which is critical for cell performance under salinity [11,12]. Tolerant plants use ions as an alternative to organic compounds for osmotic modification, which requires the synthesis of more energy (ATP) [13].

Moreover, osmolytes also protect plant cells, as they act as antioxidants, buffer the cellular redox potential, stabilize membranes and macromolecules, and function as immediate sources of energy during recovery from stress [14], which maintains the functional balance of the cell [15]. Furthermore, defense through protective enzymes superoxide dismutase, peroxidase, and catalase against salt-induced ROS over-generation and membrane lipid peroxidation is attributed to the protection of cellular membranes, which leads to salt tolerance [16].

Under salinity stress, plants adapt by initiating multiple moleculeare and physiochemical changes, which results in modifications to metabolic pathways to reach a new homeostatic equilibrium [17]. In *Brassica napus* L., higher salt stress decreases the germination parameters and biomass during the early seedling stage [18]. Additionally, in *Brassica* spp., salinity decreases nutrient absorption [19], electrolyte leakage, biomass, RWC [18,20], root length, total chlorophyll content, hypocotyl, and leaf growth with increasing POD activity and IAA oxidase [21].

Furthermore, salinity is assumed to activate the alternative gene expression patterns, which may synthesize, degrade, or decorate the metabolites from related pathways. The process is attributed to retrotransposon mobilization over-inducing salt-induced transcription factors, binding the promoter of special retrotransposons [14]. Moreover, retrotransposition bursts were reported to be critical for the reformation of gene regularity networks and for creating new metabolite patterns to tolerate and adapt to salinity stress [12]. Measuring the metabolite patterns is very important to understand the physiological and molecular responses of plants under salinity in order to illustrate the functions of genes as vital tools in functional genomics and systems biology to develop new breeding and selection strategies to improve salt tolerance in crops [6].

In the long term, metabolic disturbances are beneficial to plants, as plants use them as adaptive mechanisms, but an imbalance in Na^+^ and Cl^−^ levels in the metabolic compartments becomes toxic. The isolation of ions in the vacuoles expresses one mechanism for avoiding Na^+^ and Cl^−^ toxicity, and the levels in the leaves increase over time [22]. Previous studies were conducted on the metabolic contribution of the stress responses in rice, maize, wheat, and barley [23]. During grain filling in wheat, heat stress was shown to increase sucrose and reduced sugar phosphates and starch [24], and reducing sugar and sucrose was shown to cause reduced starch and rice seed weight [25].

During plant growth, several changes have been noted, such as metabolite changes, which can be correlated with physiological and environmental responses and genetic perturbations [23]. The significance of metabolite accumulation, such as amino acids, indicates general cellular damage in salt-sensitive cultivars. In contrast, salt-tolerant barley cultivars were shown to have an accumulation of organic acids, polyols, hexose phosphates, sugars, and tricarboxylic acid cycle (TCA) intermediates under salt stress [26].

Identifying the key metabolites and gaining a comprehensive understanding of salt-related antioxidant responses can improve the selection of desirable phenotypes of salt tolerance. Therefore, we aimed to identify metabolites that could work as biomarkers for tolerant and sensitive rapeseed cultivars that are differently adapted to salinity stress by appllying metabolite profiling and examining the diverse salinity tolerance ability of five common rapeseed cultivars through changes in their morpho-physiological traits. Our investigation contributes to the understanding of various metabolic components and morpho-physiological attributes in salinity tolerance during the early seedling stage, which can be used for further analysis.

## 2. Materials and Methods

### 2.1. Determination of Optimum Salt Stress Concentration

Five cultivars with diverse genetic backgrounds developed at the Oil Crops Research Institute, Chinese Academy of Agricultural Science, Huazhong Agriculture University, Wuhan, China, with ≥90% seed viability (Yangza 11, Zhongshuang 11, Huayouza 62, Fengyou 520, and Yangyou 9), were used in this study to determine the optimum salt concentration among concentrations of 50, 100, 150, and 200 mM L^−1^ NaCl, along with a control group (CK). Seeds were sterilized with 70% ethanol for 5 min and washed with ddH_2_O 3–5 times. The sterilized seeds were dried with blotted paper and kept at room temperature for complete redrying. Fifty uniform and healthy seeds (to decrease errors in seed germination and seedling vigor) were selected from each of the 5 cultivars and sown in germination boxes (3 technical replications with 4 biological replications) with a triple layer of germination paper containing 15 mL of NaCl solution (50, 100, 150, and 200 mM L^−1^ NaCl) or 15 mL ddH_2_O (CK). The seeds were cultured under optimal conditions (day/night temperature at 25 ± 1/20 ± 1 °C) with 12 h light (13,000 lx) and 12 h dark (HP250GS-C, Ruihua Instrument and Equipment Co., Ltd., Wuhan, China). Seed germination was recorded daily and seeds were considered to be germinated when the radical length was ≥ 2 mm. All samples were collected on the seventh day of sowing in 3 replicates, then kept at −80 °C for further analysis.

### 2.2. Phenotypic Trait Measurement

After the seventh day of germination, the final germination percentage (FG%), germination rate (GR), vigor index I (VI (I)), and vigor index II (VI (II)) were measured according to the equations reported in [27] as follows:

FG% = (*n*/*n_t_*) × 100,

where *n* is the number of germinated seeds at the end of the experiment and *n_t_* is the total number of seeds.

GR = (*a*/1) + (*b* − *a*/2) + (*c* − *b*/3) + … + (*n* − *n* − 1/*n*)

where *a*, *b*, *c*, …, *n* are numbers of germinated seeds after 1, 2, 3, …, *N* days from the start of imbibition.

VI (I) = FG% × seedling length;

VI (II) = FG% × seedling fresh weight;

$$\mathrm{Seedling}\;\mathrm{fresh}\;\mathrm{weight}\;\mathrm{stress}\;\mathrm{index}=\frac{\mathrm{Seedling}\;\mathrm{fresh}\;\mathrm{weight}\;\mathrm{stressed}}{\mathrm{Seedling}\;\mathrm{fresh}\;\mathrm{weight}\;\mathrm{nonstressed}}\times 100;$$

(1) $$\mathrm{Seedling}\;\mathrm{length}\;\mathrm{stress}\;\mathrm{index}\;=\frac{\mathrm{Seedling}\;\mathrm{length}\;\mathrm{stressed}}{\mathrm{Seedling}\;\mathrm{length}\;\mathrm{nonstressed}}\times 100.$$

After 7 days of treatment, seedlings of all cultivars were harvested, and 50 random seedlings were used to measure shoot and root length by ImageJ software. Then, the fresh and dry biomass were measured from the same seedlings after removing surface water by blotting, using 10 seedlings in each replicate according to [28].

### 2.3. Estimation of Photosynthetic Pigments, Total Soluble Sugar, and Protein Contents

Chlorophyll a (chl a), chlorophyll b (chl b), and carotenoids in fresh leaves were determined after 7 days of treatment. First, 0.5 g of fresh leaf tissue was ground and mixed with 10 mL of 80% acetone, then incubated in the dark at room temperature overnight. The absorption values of the extract at 665, 649, and 470 nm were measured using an ultraviolet spectrophotometer (UV-2100, UNIC, Shanghai, China). The contents of chl a, chl b, and carotenoids were measured according to the equations presented in [29]. Total soluble sugar content was analyzed using the anthrone sulfuric acid method. The absorbance of the samples and the standard solution was determined at 620 nm, while protein content was assessed by using bovine serum albumin as a standard, and the absorbance was estimated at 595 nm, accordingly [30]. 

### 2.4. Malondialdehyde Analysis and Proline Content

Malondialdehyde (MDA) content represents lipid peroxidation, which was measured according to [31]. In this investigation, 0.5 g of fresh shoots was homogenized in 5 mL of 10% TCA and 0.65% of 2-thiobarbituric acid (TBA). Afterward, the mixture was heated for 1 h at 100 °C and cooled at room temperature, followed by centrifugation at 10,000 rpm for 10 min. The absorbance was quantified at 450, 532, and 600 nm. Proline content was estimated by the indene triketone method [32]. Then, 0.5 g of fresh shoots was ground by a rapid automatic sample grinding instrument (JXFSTPRP24, Shanghai Jingxin Industrial Development, Shanghai, China), followed by digestion in 5 mL of 3% aqueous sulfosalicylic acid (3 g/100 mL ddH_2_O). Afterward, 2 mL of extract solution was mixed with 2 mL ninhydrin reagent and 2 mL glacial acetic acid, then boiled at 100 °C for 60 min, and the reaction was stopped by placing in an ice bath for 5 min. The mixture was extracted with 4 mL toluene and mixed vigorously using a vortex for 15–20 s, then cooled at room temperature. Free toluene was measured at 520 nm with a spectrophotometer (Beckman Coulter Inc., Fullerton, CA, USA).

### 2.5. Histochemical Analysis of O_2_^−^ and H_2_O_2_

The accumulated H_2_O_2_ and O_2_^−^ were recognized by staining leaves in 3,3-diaminobenzidine (DAB) and nitro blue tetrazolium (NBT) solution, respectively. Both dyes, with a weight of 0.025 g, were dissolved in 50 mL phosphate-buffered saline (PBS) and allowed to incubate for 2 h with slow shaking. Using a high-powered microscope attached to a high-resolution digital camera (Leica DM—2500), DAB and NBT stained leaves were photographed according to [33], and images were quantified using ImageJ (http://www.imagej.nih.gov/ij/) according to [34].

### 2.6. Measurement of Antioxidant Enzyme Activity

To determine the enzyme activity, leaf samples were ground using a rapid automatic sample grinding instrument (JXFSTPRP24, Shanghai Jingxin Industrial Development, Shanghai, China), and 0.5 g FW was homogenized in 8 mL of 50 mM potassium phosphate buffer (PPB) (pH 7.8) under cooling conditions. This homogenized solution was centrifuged at 10,000 rpm for 20 min at 4 °C, and a crude enzyme extract was obtained to measure SOD, POD, APX, and CAT according to [35,36].

Superoxide dismutase (SOD; EC 1.15.1.1) activity was determined by inhibiting photochemical reduction by nitro blue tetrazolium (NBT). The reaction mixture comprised 50 mM PPB (pH 7.8), 13 mM methionine, 75 mM NBT, 2 mM riboflavin, 0.1 mM EDTA, and 0.1 mL of enzyme extract in a 3 mL volume. One unit of SOD activity was measured as the amount of enzyme required to cause 50% inhibition of NBT reduction measured at 560 nm.

To assay peroxidase (POD; EC 1.11.1.7) activity, 0.1 mL enzyme extract was mixed with 50 mM PPB (pH 7.0), 1% (*m*/*v*) guaiacol, and 0.4% (*v*/*v*) H_2_O_2_. The absorbance was quantified at a wavelength of 470 nm.

The assay for ascorbate peroxidase (APX; EC 1.11.1.11) was done by a reaction mixture (3 mL) containing 100 mM phosphate (pH 7), 0.1 mM EDTA-Na2, 0.3 mM ascorbic acid, 0.06 mM H_2_O_2_, and 0.1 mL enzyme extract. The change in absorption was read at 290 nm for 30 s after the addition of H_2_O_2_.

The method to measure catalase (CAT; EC 1.11.1.6) activity used H_2_O_2_ (extinction co-efficient 39.4 mM^−1^ cm^−1^), 3 mL reaction mixture containing 50 mM PPB (pH 7.0), 2 mM EDTA-Na2, 10 mM H_2_O_2_, and 0.1 mL enzyme extract measured at 240 nm.

### 2.7. Determination of Na^+^ and K^+^ in Leaves

The determination of Na^+^ and K^+^ in rapeseed seedlings was carried out by following the method of [37] using a flame photometer (FP6431,Shanghai Yidian Analysis Instrument Co., Ltd. Shanghai, China). The dried powder of shoot samples (0.1 g) was digested with 2 mL of H_2_SO_4_-H_2_O_2_ mixture, filtered, and then diluted with ddH_2_O. The acid mixture (2 mL) containing ddH_2_O was considered blank. A standard curve of Na^+^ and K^+^ (10–100 µg mL^− 1^) was used as a reference.

### 2.8. Metabolite Extraction and Detection

Seeds were collected after 12 and 24 h after being sown under salt treatment. Freeze-dried seeds were crushed using a mixer mill (MM 400, Retsch GmbH, Haan, Germany), and the powder was weighed and extracted overnight at 4 °C with 1.0 mL of 70% aqueous methanol containing 0.1 mg L^−1^ lidocaine for water-soluble metabolites. Following centrifugation at 10,000 rcf for 10 min, the extracts were absorbed (CNWBOND Carbon-GCB SPE Cartridge, 250 mg, 3 mL; ANPEL, Shanghai, China, www.anpel.com.cn/cnw) and filtrated (SCAA-104, 0.22 mm pore size; ANPEL http://www.anpel.com.cn/) before LC-MS analysis. Samples were subjected to metabolite detection using high-performance liquid chromatography (HPLC) and high-performance liquid chromatography mass spectrometry (LC-MS/MS) [38]. The relative contents of each of these 332 identified metabolites were quantified using the scheduled multiple reaction monitoring (sMRM) method described previously [39]. The sMRM algorithm was used with an MRM detection window of 90 s and a target scan time of 1.0 s using Analyst 1.5 software. Given that biological variance is considerably higher than technical variance, we chose not to carry out technical replication. The analytical conditions were taken from a previous report [40] with minor modifications as follows: HPLC: column, shim-pack VP-ODS C18 (pore size 5.0 μm, length 2 × 150 mm); solvent system, water (0.04% acetic acid): acetonitrile (0.04% acetic acid); gradient program, 95:5 *v/v* at 0 min, ramping to 0:100 *v/v* at 15 min, 0:100 *v/v* at 15–17 min, 95:5 *v/v* at 17–17.1 min, 95:5 *v/v* at 17.1–22 min; flow rate, 0.35 mL min^–1^; temperature, 40 °C; injection volume: 5 μL for one run.

### 2.9. Metabolite Analysis and Identification

The metabolite fold changes (FCs) were calculated and volcano plots were generated using MetaboAnalyst [23]. An increase in FC (ratio of metabolites in salt-stressed samples to control samples) was considered significant when FC ≥ 1 and the concentration of the metabolite significantly different (*p* < 0.05) between control and stressed plants. Metabolites were identified by comparing with the standards (wherein the “identifications” column was labeled as “standard”) or taken from previous experiments (labeled as “putative”) (supplementary data file 2) [41]. The metabolic pathway map was constructed based on the relevant literature and the KEGG database (https://www.kegg.jp/kegg-bin/show_pathway?161345586717248/vvi01100.args, accessed on 1 March 2021).

### 2.10. Statistical Analysis

The phenotypic data collected from the experiment were analyzed statistically. Two-way analysis of variance (ANOVA) of germination and seedling traits for all accessions was conducted using the Statistix 8.1 software package. The significance of differences between groups was further validated and determined by Duncan’s multiple range test (DMRT) at a significance level of *p* < 0.05. The graphical representation was constructed using GraphPad prism (V: 8.0.1) (RStudio software, San Diego, CA, USA). The standard error is mentioned in the figures.

## 3. Results

### 3.1. Impact of NaCl Treatment on Germination Parameters, Phenotypic Appearance Traits, and Vegetative Biomass of Five Rapeseed Cultivars

**Figure 1:**
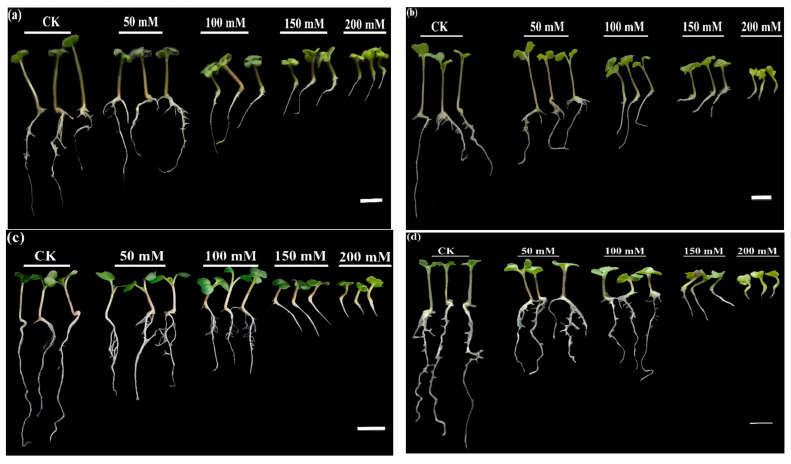

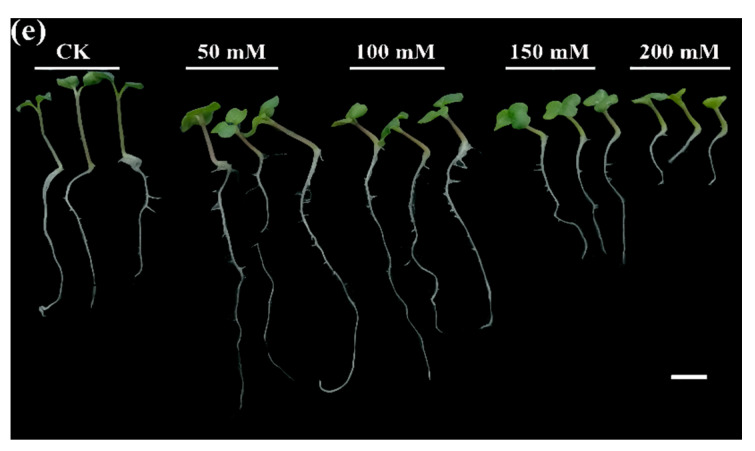
Impact of NaCl treatment on seedling growth of (**a**) Yangza 11, (**b**) Zhongshuang 11, (**c**) Huayouza 62, (**d**) Fengyou 520, and (**e**) Yangyou 9 rapeseed cultivars during the early seedling stage. Scale bar: 1 cm.

To estimate the different responses of five common rapeseed cultivars to salinity stress during the seed germination and early seedling growth stage, we investigated the germination and growth parameters under various concentrations of NaCl (0, 50, 100, 150, and 200 mM L^−1^) during 7-day treatments. Based on the morphological analysis (Table S1, Figures S1–S3), we used 150 mM NaCl to complete the study and evaluate the salt influence on salt-tolerant and -sensitive rapeseed cultivars using physiochemical parameters. Among the studied cultivars, Zhongshuang 11 displayed the lowest growth rate (Figure 1b), while Yangyou 9 showed higher growth (Figure 1e) compared to other cultivars with 150 and 200 mM of NaCl.

### 3.2. Alterations in Photosynthetic Pigments under Salt Stress

**Figure 2:**
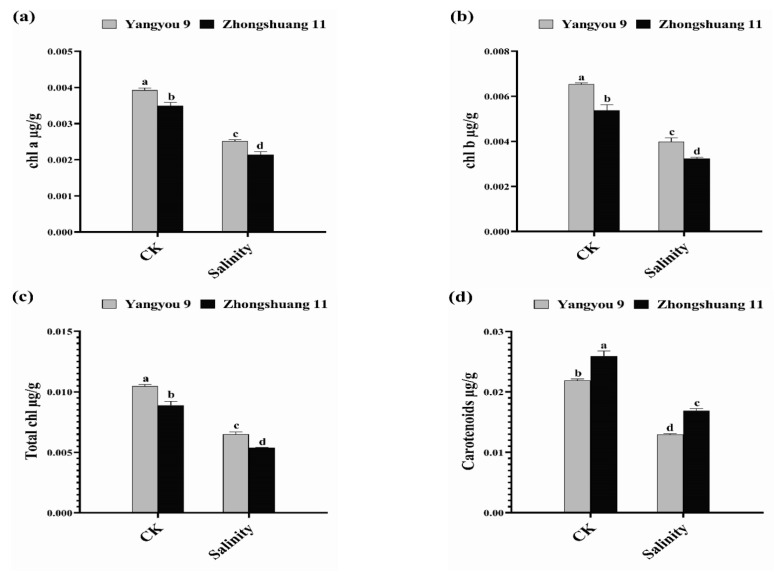
Modifications in (**a**) chlorophyll a, (**b**) chlorophyll b, (**c**) total chlorophyll, and (**d**) carotenoid content under normal and salt conditions induced by 150 mM NaCl. Bars represent ±SE of three replicates. Letters (a, b, c and d) on vertical bars represent significant differences between cultivars and treatments according to Duncan’s multiple range test (DMRT) at *p* < 0.05.

Salt stress caused a marked dose-dependent decline in photosynthetic pigments (chl a, chl b, and carotenoids) in both rapeseed cultivars. The response of the two cultivars with regard to photosynthetic pigments was not the same under 150 mM NaCl: the salt-induced decrease in photosynthetic pigments was 36.05 and 38.88% (chl a), and 39.26 and 39.77% (chl b) in Yangyou 9 and Zhongshuang 11, respectively (Figure 2a,b). A similar trend was observed for total chlorophyll in both cultivars; in Yangyou 9 and Zhongshuang 11, it decreased by 38.06 and 39.42%, respectively, under 150 mM NaCl versus normal conditions (CK) (Figure 2c). Additionally, salt caused significant perturbations in carotenoids in Yangyou 9 and Zhongshuang 11, which were reduced by 40.58 and 34.89%, respectively, under stress conditions versus CK (Figure 2d).

### 3.3. Alterations in Total Soluble Sugar, Total Soluble Protein, MDA, and Proline Content under Salt Stress

**Figure 3:**
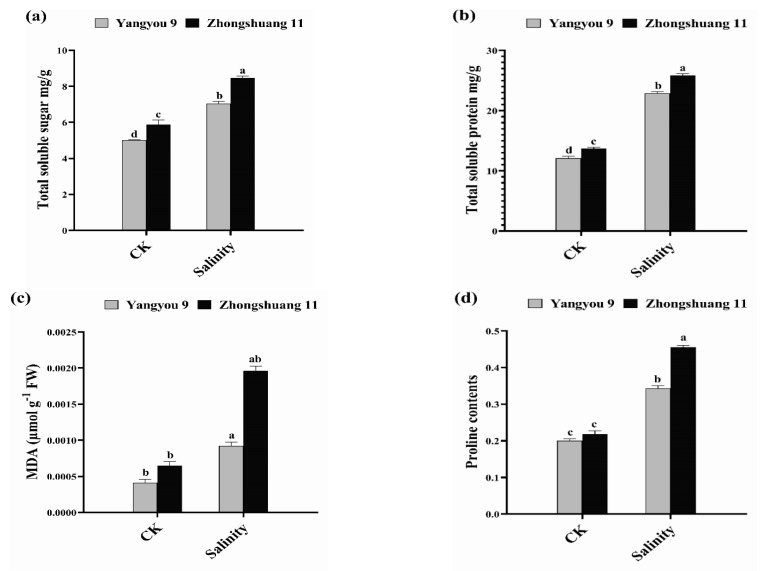
Modifications in (**a**) total soluble sugar, (**b**) total soluble protein, (**c**) MDA, and (**d**) proline content under normal and salt conditions induced by 150 mM NaCl. Bars represent ±SE of three replicates. Letters (a, b, c and d) on vertical bars represent significant differences between cultivars and treatments according to Duncan’s multiple range test (DMRT) at *p* < 0.05.

Total soluble sugar (TSS) and protein (TSP) are the most significant osmolytes that actively participate in osmoregulation under stress conditions. Unstressed Zhongshuang 11 seedlings showed higher TSS and TSP content of 5.88 and 13.69 mg/g, while Yangyou 9 recorded values of 5.01 and 12.09 mg/g, respectively. However, salinity stress increased TSS and TSP content by 40.68 and 89.04% (Yangyou 9) and 43.96 and 88.89% (Zhongshuang 11), respectively, versus CK (Figure 3a,b).

On the other hand, under normal growth conditions (CK), Zhongshuang 11 showed slightly higher MDA and percent of proline content (0.0009 µmol g^−1^ FW and 0.219%) compared to Yangyou 9 (0.0004 µmol g^−1^ FW and 0.200%). Notably, salt stress increased MDA and proline content by 123.7 and 71.35% (Yangyou 9) and 201.6 and 108.4% (Zhongshuang 11), respectively (Figure 3c,d).

### 3.4. Accumulation of O_2_^−^ and H_2_O_2_ under Salt Stress in Yangyou 9 and Zhongshuang 11 

**Figure 4:**
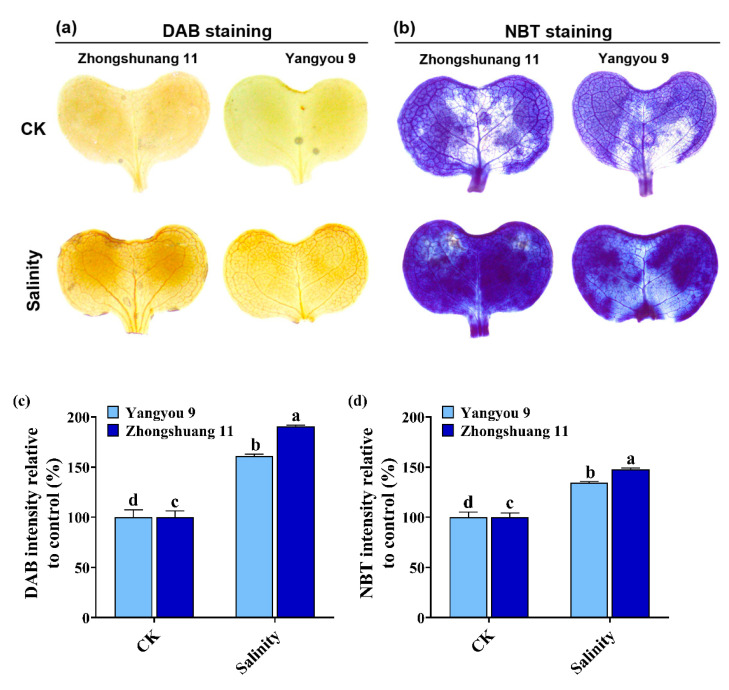
ROS accumulation in Yangyou 9 and Zhongshuang 11 leaves with (**a**) 3, 3-diaminobenzidine (DAB), (**b**) nitro blue tetrazolium (NBT), (**c**) DAB intensity relative to control (%), and (**d**) NBT intensity relative to control (%) under NaCl (150 mM). Bars represent ±SE of three replicates. Letters (a, b, c and d) on vertical bars represent significant differences between cultivars and treatments according to Duncan’s multiple range test (DMRT) at *p* < 0.05.

After 7 days of salt treatment, H_2_O_2_ and O_2_^–^ accumulation was examined in leaves of rapeseed seedlings. Histochemical detection of ROS (O_2_^−^ and H_2_O_2_) using NBT and DAB staining revealed that seedlings subjected to salt stress accumulated larger amounts of ROS. Our results show weaker staining in Yangyou 9 leaves (tolerant cultivar) for both O_2_^−^ and H_2_O_2_ under stress compared with Zhongshuang 11 (sensitive cultivar), with the salinized leaves of the latter being more damaged than leaves of Yangyou 9 seedlings (Figure 4a,b). Additionally, DAB and NBT intensity was higher under salt stress relative to control (%), which was higher in Zhongshuang 11 (190.4 and 147.8%, respectively) as compared to Yangyou 9 (161.1 and 134.2%) (Figure 4c,d).

### 3.5. Alterations in Antioxidant Enzyme Activity under Salt Stress

**Figure 5:**
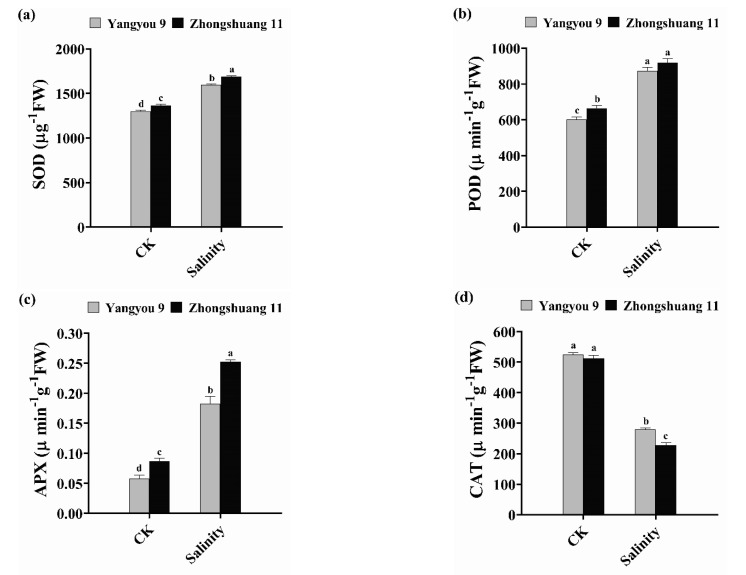
Modifications in (**a**) superoxidase (SOD), (**b**) peroxidase (POD), (**c**) ascorbate peroxidase (APX), and (**d**) catalase (CAT) activity under normal and salt conditions induced by 150 mM NaCl on fresh samples. Bars represent ±SE of three replicates. Letters (a, b, c and d) on vertical bars represent significant differences between cultivars and treatments according to Duncan’s multiple range test (DMRT) at *p* < 0.05.

Adding NaCl to the growth medium resulted in a marked change in antioxidant enzyme activity in both rapeseed cultivars (Yangyou 9 and Zhongshuang 11). Salt stress caused marked dose-dependent changes in antioxidant enzyme activity (SOD, POD, APX, and CAT). Under normal conditions, Yangyou 9 recorded values of 1464 µg^−1^ FW, 601.2 µmin^−1^ g^−1^ FW, 0.058 µmin^−1^ g^−1^ FW, and 535.3 µmin^−1^ g^−1^ FW, while Zhongshuang 11 recorded values of 1400 µg^−1^ FW, 664.6 µmin^−1^ g^−1^ FW, 0.087 µmin^−1^ g^−1^ FW, and 552.6 µmin^−1^ g^−1^ FW, on SOD, POD, APX, and CAT, respectively (Figure 5a–d). The enzyme response of the two cultivars was not the same under NaCl stress: salt increased SOD, POD, and APX by 15.27, 45.46, and 214.8% in Yangyou 9 and by 13.96, 38.21, and 191.1% in Zhongshuang 11, respectively. Meanwhile, CAT activity decreased under salinity stress by 53.40 and 58.75% in Yangyou 9 and Zhongshuang 11, respectively, versus CK (Figure 5a–d).

### 3.6. Impact of NaCl on Na^+^, K^+^, and Na^+^/K^+^ Ratio in Shoots 

Under salinity stress, Na^+^ uptake was decreased, and K^+^ uptake was increased in the tolerant cultivar (Yangyou 9) compared to the sensitive cultivar (Zhongshuang 11). Under normal conditions, Na^+^ content decreased by 24.95% and K^+^ content increased by 16.39% in the Yangyou 9 versus Zhongshuang 11 shoots. Meanwhile, unstressed Zhongshuang 11 seedlings showed higher Na^+^ content of 48.76 mg/g and lower K^+^ content of 4.87 mg/g, while Yangyou 9 recorded values of 30.47 and 6.83 mg/g, respectively. On the other hand, the Na^+^/K^+^ ratio in Yangyou 9 shoots decreased by 35.52% (normal conditions) and 55.48% (stress conditions) compared to Zhongshuang 11 (Table 1).

### 3.7. Relationships and Variation among Growth and Biochemical Attributes of Two Rapeseed Cultivars

**Figure 6:**
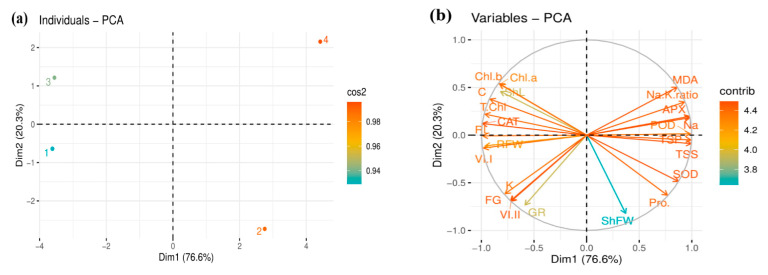
Principal component analysis (PCA) of salt treatment and relationships of variable traits in two rapeseed cultivars: (**a**) PCA score plot of salt treatment on rapeseed seedlings, and (**b**) PCA loading plot of PC1 and PC2 of examined variable traits; circles indicate most correlated variables. Score plot represents separation of treatments as (1) Ck and (2) salinity treatment for Yangyou 9, and (3) CK and (4) salinity treatment for Zhongshuang 11. Tested variables include final germination (FG %); germination rate (GR); vigor index I (VI (I)); vigor index II (VI (II)); shoot length (ShL); root length (RL); shoot fresh weight (ShFW); root fresh weight (RFW); chlorophyll a (Chl a); chlorophyll b (Chl b); total chlorophyll (T. Chl); carotenoid (C) content; total soluble sugar (TSS); total soluble protein (TSP); proline (pro. %); malondialdehyde (MDA) content; sodium ions (Na^+^); potassium ions (K^+^); superoxidase (SOD) activity; peroxidase (POD) activity; ascorbate peroxidase (APX) activity, and catalase (CAT) activity.

The score and loading plots of principal component analysis (PCA) were used to evaluate the performance of two *B. napus* cultivars under salt stress (150 mM NaCl). All 24 traits were loaded into two major principal components, PC1 (Dim1) and PC2 (Dim2), which showed a cumulative variance of about 96.9% in the dataset, where PC1 explained 76.6% of variation and PC2 revealed the difference of 20.3%, indicating variation of salt treatment applied on rapeseed. The distribution of the two cultivars displayed a clear signal of salinity stress, indicating significant effects on the studied characteristics of rapeseed. Specifically, Yangyou 9 was more displaced than Zhongshuang 11 under salt conditions, indicating that Yangyou 9 could alleviate the salt toxicity on the seeds and enhance their germination and early seedling growth (Figure 6a).

The PCA loading plot shows clear visualization and variation of the studied growth-related parameters. The members of the first group of variables with PC1 (ShFW, proline, SOD, TSS, TSP, Na^+^, POD, APX, Na^+^/K^+^, and MDA) are positively correlated with each other but negatively correlated with chl, ShL, RL, RFW, GR, VI (I), VI (II), FG%, and GR. In contrast, a positive correlation is noticed in the remaining attributes, aligned with PC2: chl, GR, VI (I), RFW, ShL, and RL (Figure 6b).

### 3.8. Metabolic Changes of Yangyou 9 (T) and Zhengsheng 11 (S) in Response to Salt Stress 

For further clarification, the physiological mechanisms of salt tolerance underlying the salt-tolerant Yangyou 9 and Zhongshuang 11 metabolic changes during the early germination stage (12 and 24 h after sowing) were studied under salinity stress (150 mM NaCl) compared to control (no NaCl). Using a metabolomic approach based on HPLC-QQQ mass spectrometry, 332 compounds were detected. Statistical analysis was conducted to minimize the data complexity and get significant differences. The metabolites list came from [41]. A total of 225 metabolites were filtrated according to *p* < 0.05, as shown in Table S2. According to heatmap, principal component analysis (PCA), and cluster-based analysis, there were clear distinctions between samples within treatments and genotypes. All replications within each treatment clustered together, indicating that the changes induced by salinity were hierarchically greater than biological and technical variability (Figure S4a–d).

**Figure 7:**
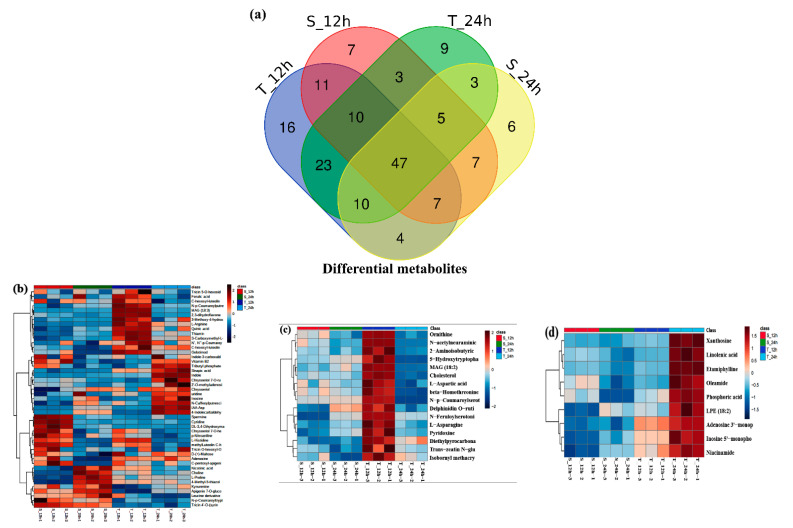
Differential metabolites of Yangyou 9 (T) and Zhengsheng 11 (S), fold change >1. (**a**) Venn diagram and (**b**) heatmaps of differential metabolites during 12 and 24 h of germination under salt treatment for tolerant (T) and sensitive (S) cultivar. Heatmaps of differential metabolites in tolerant cultivar after (**c**) 12 h and (**d**) 24 h of germination under salt treatment.

To further clarify the differential metabolites between Yangyou 9 (T) and Zhengsheng 11 (S) during 12 and 24 h of germination under salinity stress (150 mM NaCl), a Venn diagram was drawn to illustrate discriminating metabolites common to and distinct between the two cultivars (Figure 7a) by fold change (FC  >  1) and Student’s t-test (*p* < 0.05). Our results show that the accumulation of 47 metabolic compounds was involved in responding to salt stress in the two cultivars during the studied times (12 and 24 h) (Figure 7b). Interestingly, Yangyou 9, the tolerant cultivar, is characterized by accumulating some important metabolic compounds; 16 metabolic compounds were accumulated after 12 h: MAG (18:2), cholesterol, L-aspartic acid, L-asparagine, ornithine, beta-homothreonine, 5-hydroxytryptophan, N-p-coumaroylserotonin, N-feruloylserotonin, trans-zeatin N-glucoside, pyridoxine, delphinidin O-rutinoside, N-acetylneuraminic acid, isobornyl methacrylate, 2-aminoisobutyric acid, and diethylpyrocarbonate (Figure 7c). In comparison, nine metabolite compounds were accumulated after 24 h: LPE (18:2), linolenic acid, xanthosine, inosine 5’-monophosphate, adenosine 3’-monophosphate, niacinamide, oleamide, phosphoric acid, and etamiphylline (Figure 7d), indicating that they might be involved in enhancing salt tolerance. Heatmaps of the normalized intensity of these metabolites are presented in Figure 7b–d.

#### 3.8.1. Amino and Polyamine-Related Metabolites 

We found an increase in some amino acids and their derivatives, the most important of which were L-histidine, L-arginine, and L-proline, which increased by 1.43-, 1.74-, and 1.11-fold (12 h) and 1.08-, 1.38-, and 1.11-fold (24 h) in Yangyou 9, and by 1.37-, 1.13-, and 1.07-fold (12 h) and 1.06-, 1.15-, and 1.17-fold (24 h) in Zhongshuang 11. Moreover, a leucine derivative, kynurenine, and N-p-coumaroyltryptamine were increased in both cultivars under salt treatment during 12 and 24 h of seed germination; we noted an increase in the accumulation of serotonin and its derivatives. Of note, it increased the accumulation of trigonelline and betaine (alkaloid compounds) by 1.26- and 1.10-fold (12 h) and 1.15-, and 1.05-fold (24 h) in Yangyou 9, and decreased it in Zhongshuang 11. However, spermine (polyamine) increased by 1.20- and 1.55-fold (12 h) and 1.23-, and 1.25-fold (24 h) in Yangyou 9 and Zhongshuang 11, respectively (Table S2).

#### 3.8.2. Polyphenolic-Related Metabolites 

Phenolic compounds were altered in salt-treated Yangyou 9 and Zhongshuang 11 cultivars; most of them were highly accumulated. Alterations in polyphenol compounds were evident during the time of germination. Quinic acid, sinapic acid, and ferulic acid were accumulated in both Yangyou 9 and Zhongshuang 11 at 12 and 24 h of germination, while catechin and N-feruloylserotonin were increased only in a Yangyou 9 (tolerant cultivar) at both times. Moreover, coniferyl aldehyde increased during both times, while benzamidine accumulated only at 24 h of seed germination in Zhongshuang 11. In addition, phenol amine compounds such as N-caffeoylputrescine, N-p-coumaroylputrescine, and N’, N’’-p-coumaroyl feruloylspermidine accumulated in the two cultivars, whereas p-coumaroy l-2-hydroxyputrescine accumulated at 12 h of germination, then decreased at 24 h. Additionally, N’, N’’-di-p-coumaroylspermidine increased in Zhongshuang 11 only during 12 and 24 h of seed germination (Table S2).

#### 3.8.3. Flavonoid-Related Metabolites

There was a significant change in the accumulation of flavonoid compounds based on the time of seed germination and the cultivar, and there was a clear difference between 12 and 24 h of germination and increased accumulation of flavonoid compounds in Yangyou 9 (tolerant cultivar) compared to Zhongshuang 11 (sensitive cultivar) during those times. The amounts of several flavonoids (apigenin 7-O-glucoside, C-pentosyl-apigenin O-hexoside, C-hexosyl-luteolin O-hexoside, C-hexosyl-luteolin O-p-coumaroylhexoside, chrysoeriol 7-O-hexoside, chrysoeriol 7-O-rutinoside, tricin O-hexosyl-O-hexoside, and tricin 4’-O-(syringyl alcohol) ether 5-O-hexoside, and methyl luteolin C-hexoside) were enhanced under salt stress in both cultivars at both time points. Meanwhile, some flavonoids (chrysoeriol C-hexoside, C-pentosyl-apeignin O-feruloylhexoside, luteolin 6-C-glucoside, tricin 7-O-hexoside, and chrysoeriol C-hexoside) showed an opposite trend. However, the level of chrysoeriol was accumulated in the tolerant and sensitive cultivar, but was higher in the former. Some flavonoids (chrysoeriol O-malonylhexoside, tricin, Selgin 5-O-hexoside, and cyanidin 3,5-di-O-hexoside) showed accumulation in Yangyou 9 and alleviation in Zhongshuang 11 under stress at both 12 and 24 h of seed germination under salt treatment (Table S2).

Interestingly, vitamin B2 was accumulated by 1.23-fold in Zhongshuang 11 after 12 h, whereas it increased by 1.20-fold in Yangyou 9 after 24 h. Moreover, pyridoxine O-glucoside, thiamin, 4-methyl-5-thiazoleethanol, and choline accumulated by 1.28-, 1.21-, 1.26-, and 1.23-fold at 12 h and 1.18-, 1.09-, 1.23-, and 1.17-fold at 24 h in Yangyou 9, whereas it decreased in Zhongshuang 11 by 1.08-, 1.04-, 1.02-, and 1.05-fold at 12 h and 0.80-, 1.07-, 1.43-, and 1.48-fold at 24 h. Additionally, the accumulation of carbachol and niacinamide was increased by 1.24- and 1.15-fold after 24 h in Yangyou 9, higher than in Zhongshuang 11 (Table S2).

#### 3.8.4. Carbohydrate-Related Metabolites 

In this investigation, there was increased sugar compound accumulation during seed germination in the studied cultivars. We found increases in fructose 1, 6-diphosphate, sucrose, D-(+)-maltose, and α-lactose by 1.20-, 1.05-, 2.56-, and 1.08-fold at 12 h and 1.16-, 1.06-, 2.59-, and 1.06-fold at 24 h in Yangyou 9. All compounds were decreased after 12 h in Zhongshuang 11 except for D-(+)-maltose, which increased by 2.58-fold at 12 h and 2.40-fold at 24 h and fructose 1, 6-diphosphate and sucrose by 1.13-, and 1.05-fold at 24 h. In addition, a-L-rhamnose was decreased in both cultivars at 12 and 24 h of seed germination under salt treatment. On the other hand, we noticed an increase in polygodial (terpene compounds) at 12 and 24 h by 1.33- and 1.10-fold, respectively, in Yangyou 9 and a decrease in Zhongshuang 11; additionally, diosgenin was increased at 12 and 24 h in Zhongshuang 11 and increased only at 24 h in Yangyou 9 (Table S2).

#### 3.8.5. Fatty Acid-Related Metabolites in the Two Cultivars

Fatty acid content was decreased at 12 and 24 h of seed germination in response to salinity stress, and the decrease was more significant in the sensitive cultivar (Zhongshuang 11) than the tolerant cultivar (Yangyou 9). Among fatty acid compounds, MAG (18:3) was increased by 1.47- and 1.05-fold at 12 h and 1.08- and 1.07-fold at 24 h in Yangyou 9 and Zhongshuang 11, respectively. Furthermore, the accumulation of some fatty acids increased after 24 h of germination in the resistant cultivar, including LPE (18:2), linolenic acid, and 14,15-dehydrocrepenynic acid, while there was an increase in lysoPE 18:2 only at 24 h of germination in the sensitive cultivar (Table S2).

#### 3.8.6. Nucleic Acid-Related Metabolites 

The resistant cultivar showed a noticeable change in accumulation of nucleic acid and its derivatives at 12 and 24 h of seed germination under salt treatment, including crotonoside, guanosine, N2, N2-dimethylguanosine, and N-(9H-purin-6-ylcarbamoyl) threonine. In contrast, uridine, inosine, uracil, cytidine, and adenosine were accumulated in both cultivars (Table S2).

#### 3.8.7. Other Metabolites 

Several other metabolites that fall into a variety of biochemical classes were altered in the two cultivars: p-Nitroaniline, 2’-O-methyladenosine, golotimod, 1-[5-(2,3,4-trihydroxybutyl)-2-pyrazinyl]-1,2,3,4-butanetetrol, DL-3,4 dihydroxymandelic acid, S-carboxymethyl-L-cysteine, 2,3-dihydroflavone, 3-methoxy-4-hydroxybenzoic acid O-hexoside, 4-Indolecarbaldehyde, and tributyl phosphate. These were significantly enhanced under salinity stress at 12 and 24 h. In addition, hinokinin showed higher levels under salt stress at 24 h in both cultivars. Many other metabolites, including DIMBOA glucoside, pinoresinol 4-O-glucoside, N-acetyl-L-2-aminoadipic acid, and 4-hydroxybenzoic acid O-hexoside, were accumulated in the salt-tolerant cultivar (Yangyou 9), and decreased in salt-sensitive cultivar (Zhongshuang 11), at 12 and 24 h of seed germination under salinity stress (150 mM NaCl) (Table S2).

Furthermore, the resistant cultivar showed a noticeable change in hormone accumulation at 12 and 24 h of seed germination under salt treatment, indicating the critical role of hormones in plant tolerance to saline stress. For example, in Yangyou 9, the accumulation of gibberellin A14 was increased by 1.07- and 1.20-fold after 12 and 24 h of germination, and we found an increase in IAA-Asp, IAA-Glu, indole, and melatonin by 1.46-, 1.13-, 1.26-, and 1.19-fold at 12 h and 2.44-, 1.07-, 1.80-, and 1.23-fold at 24 h, while melatonin was decreased in Zhongshuang 11 at 12 and 24 h. The accumulation of methoxy indoleacetic acid increased in Zhongshuang 11 only at 12 and 24 h (Table S2).

## 4. Discussion

### 4.1. Differences in Morpho-Physiological Alterations in Response to Salinity between Yangyou 9 and Zhongshuang 11

Seed germination is a very sensitive process in the plant’s life cycle, as it supports seedling development and survival, which is largely affected by genetic traits, moisture availability, and soil quality [42]. Our findings indicate that salt stress reduced the studied attributes of germination and seedling growth, especially in the Zhongshuang 11 cultivar, indicating that it was the less tolerant cultivar under high salt concentrations. Previous studies confirmed a negative relationship between salt concentration and germination parameters, ultimately leading to delayed germination in rapeseed [43], which is consistent with our study. Salinity affects the development process by causing an imbalance in the hormonal system and cellular functions of seeds, which alter enzymatic activity, changes metabolism, reduces the use of seed reserves, and reduces water uptake (osmotic effect) or ionic imbalance (ionic effect), ultimately slowing the growth rate [44,45].

It is always difficult to know whether a reduced photosynthesis rate is the cause of growth reduction or the result. Salinity causes an imbalance of chlorophyll and its intermediates (toxic photodynamic molecules that produce ROS, such as singlet oxygen), thus the chlorophyll metabolism must be strictly regulated under abiotic stresses [46]. Chloroplasts are the most important organelles responsible for photosynthesis, and an increased Na^+^ or Cl^−^ concentration under salt stress leads to an imbalance in the photosynthesis process and chlorophyll production. Thus, chlorophyll is a substantial indicator of metabolite changes in salinized plants [47]. A study on *Brassica napus* [48] explained that the growth rate decreased with increased salt concentration through an imbalance in the photosynthesis and ET rates and a decrease in PS II efficiency.

Osmotic adjustment decreased the osmotic potential by increasing external osmolality, maintaining water absorption and various physiological processes in the plants [6,49], and including total soluble sugars and proteins, proline, amino acids, glycine betaine, etc., where they reduce osmotic stress. The increased proline and total soluble sugar content plays a vital role in protecting the cells under salt stress by maintaining the osmotic pressure and ionic balance in the cytosol and outside the cell, which increases the absorption of water and nutrients and balance of relative water content, which leads to increased protein function and cellular membrane stability [50,51]. Our investigation showed that the tolerant cultivar exhibited higher proline accumulation and elevated total soluble sugar and protein under salinity stress, which is in line with [30,38,52].

Under salinity stress, there is increased accumulation of toxic ions, especially sodium ions (Na^+^), which leads to an imbalance in the ion and hyperosmosis levels in plants. As a result of this imbalance, there is weakness in the physiochemical processes of plant cells, which negatively affects development [1]. Moreover, in salinized plants with higher Na^+^ levels, K^+^ uptake is inhibited, which elevates the Na^+^/K^+^ ratio in the cellular tissues [53]. Our findings show that the tolerant cultivar worked to save and increase the K^+^ in leaves and decrease Na^+^ accumulation, which modulates Na^+^ and K^+^ uptake, to improve seedling growth, and showed an association with the restricted accumulation of toxic Na^+^ ions; thus, it reduced the generation of increased oxidative stress due to salt stress (NaCl), which agrees with the results of [54,55].

Salinized seedlings displayed excessive accumulation of H_2_O_2_, hence oxidative stress, which also resulted in higher lipid peroxidation, thereby causing leakage of cellular components [56]. Abiotic stress, especially salinity, causes oxidative damage to plants due to excessive accumulation of ROS, which increases the damage to cellular components and injures cell structure and function, including membrane lipids, DNA damage, and enzyme inactivation [57]. Plants have comprehensive antioxidative systems, which play a critical role in scavenging ROS accumulation, whereas CAT and SOD mitigate the harmful effects of oxidative stress; likewise, SOD catalyzed O_2_^•–^ is dismutated into O_2_^•–^ and H_2_O_2_ [58,59].

Furthermore, the osmotic effects of salinity lead to metabolic changes, which disrupt the plant’s ability to absorb and retain water, thereby increasing ROS levels [48]. Previous studies showed increased ROS accumulation in plants grown under salinized conditions and emphasized the role of ROS in damage to cell membranes and decreased crop yield, as it is one of the main causes of cytotoxicity under salt stress [6]. Conclusive results of a previous study in wheat and barley showed that antioxidant enzymes are involved in salt stress tolerance [60] in rapeseed [30]. Additionally, osmolytes reduced salt-induced oxidative stress by direct or indirect ROS degeneration and induced antioxidant enzyme activity [49], which agrees with our results showing that antioxidant enzymes and osmolyte accumulation were enhanced under salt stress.

### 4.2. Differences in Accumulation of Metabolites in Response to Salinity in Yangyou 9 and Zhongshuang 11

Metabolomics analysis is a helpful tool to understand the mechanisms underlying salt tolerance in plants. Recently, metabolic studies of the variations between salt-sensitive and salt-tolerant plants have revealed an interesting finding, that the levels of constitutive metabolites in tolerant varieties differ from those in sensitive varieties in the same species, and various species have shown both conserved and divergent metabolite responses. Moreover, specific metabolite synthesis is limited to a few plants. Thus, it would be helpful to perform further metabolic studies between salt-sensitive and salt-tolerant plants in the same species. In the current study, there was a clear distinction among the metabolites in response to salt stress between the two cultivars (Yangyou 9 and Zhongshuang 11) and treatments (12 and 24 h after sowing).

Under different salinity levels, there are changes in amino acid (Aa) content where the reorganization occurs, such as increased proline, amides, glutamate, and alanine content, while there are no changes in others [61]. The Aa content in this study decreased at 12 and 24 h of germination under salinity stress, and the decrease was more significant in the sensitive cultivar (Zhongshuang 11) than the tolerant cultivar (Yangyou 9); additionally, we found an accumulation of Aa derivatives that might act as compatible osmolytes under salt stress. In particular, Aas found at low concentrations in the cells increased under salinity pressure, especially branched-chain Aas, which can function as alternative electron givers in the electron transport chain and as compatible compounds in the mitochondria [62,63]. In *Arabidopsis*, after 12 h germination under salt treatment, there was an increase in the biosynthesis of aromatic Aas, which are primary factors stimulating the biosynthesis of lignin, which works to strengthen the cellular walls [64].

Osmolytes are one of the most important compatible solutes with low weight, which enables them to help remove excessive ROS and stabilize proteins by replacing water on the surface of the protein membrane [65]. The obtained results indicate an increased level of proline with increased exposure time during seed germination under salt stress, especially in Yangyou 9 (tolerant cultivar), and these results correspond to [65,66]; those studied suggested that the increased accumulation of osmolytes in the cells is associated with decreased accumulation of ROS, thus plants are better able to grow and develop under salinity stress. Fatty acid and lipid regulation are an essential means of protection in the plant under salt stress [67], as it works to maintain the safety of cellular membranes [68]. Our results show a change in the content of nitrogen-containing compounds, such as amines that relieve oxidative stress, the balance of enzymes, and the safety of cell membranes under salt stress due to increased Na^+^ and Cl^−^ levels in the cells [69]. We also noted an increased accumulation of serotonin and its derivatives, which have a known role in plant resistance to various stressors [70].

Carbohydrates act as osmolytes, maintain cellular balance under salt stress, and have an important role in a host of biological functions [70]. An increase in sugar content was observed under the salt stress (100 mM) during 1–24 h in rice tissue cultures, including fructose, phosphorylated sugars, glucose, and galactose [71], whereas, under long-term NaCl exposure, co-induction of glycolytic metabolites and sucrose in *Arabidopsis* tissue cultures was observed [72]. The content of soluble sugars and other unknown putative complex glycans increased under high salinity levels in *Arabidopsis*, *Thellungiella salsuginea*, and durum wheat. Trehalose application decreased the adverse effect of salinity in rice [38]. There is a link between increased mannitol, sorbitol, cyclic polyols, and myo-inositol, and its methylated derivatives and salt tolerance; additionally, application of trehalose and polyols prevents oxidation of salt-bound lipids and protects the cells from damage by coating large particles in a protective shell, as well as reducing ROS accumulation [73].

The polyamines (Pas) have an essential role in modulating plant defense responses to various stresses, such as salinity, and include putrescine, spermidine, and spermine (free, conjugated, and bound polyamines) [74]. Due to their antioxidant properties, the Pas play a role in stabilizing cell walls and maintaining cell membrane function, in addition to having anti-aging and anti-stress effects. Moreover, Pas regulate the processes of gene expression and the formation of ionic bonds between anions i.e., DNA, RNA, and protein. Exogenous Pas (putrescine) have positive effects under salt stress conditions, by elevating the salinity tolerance [75]. In addition, Pa conjugates such as N-caffeoylputrescine, N-p-coumaroylputrescine, and N’, N’’-p-coumaroyl feruloylspermidine affect the response to salt treatment; they increased significantly in Yangyou 9 as compared to Zhongshuang 11. Previous studies confirmed the role of these compounds in maintaining the balance of secondary metabolic pathways. They also have an influential role in the life cycle and development of plants by participating in the processes of cell division and different physiological responses under stress conditions [74,76].

Under salt stress, flavonoids are considered to be one of the most crucial plant phenolics, playing an essential role in the plant’s tolerance against salt stress, as these are potent antioxidants [66]. In this investigation, the obtained results show a difference in flavonoid content, which shows the effect of salt on compounds of flavonoids and other phenolics in rapeseed seeds. As the increase in Yangyou 9 was greater than Zhongshuang 11 at 12 and 24 h under treatment during seed germination, we agree that the increased accumulation of these compounds has a role in reducing the oxidative damage caused by increased ROS accumulation under salt stress [66,75].

Terpenes are natural plant products found in a simple, undefined form and often accumulate as accompaniments to carbohydrates. They participate in important functions such as pest resistance and contribute to crop quality and yield, and are accumulated in the epicuticular and intracuticular wax layers of stem and leaf surfaces, protecting the plant under abiotic stresses [77]. In this study, the content of terpenes in the resistant cultivar (Yangyou 9) was increased compared to the sensitive cultivar (Zhongshuang 11) during 12 and 24 h of seed germination under salt treatment. Additionally, terpenes participate in interactions with various transcription factors concerning apoptosis, cell cycle, and DNA reform due to oxidative imbalance under different abiotic stresses, especially salinity stress [70].

In this study, we found an increase in the level of plant hormones, including cytokinins (CK), gibberellins (GA), and indole acetic acid (IAA), in Yangyou 9 compared to Zhongshuang 11. The difference in GA and CK content confirms their role in different responses to oxidative stress caused by salinity stress [38]. Previous studies verified the role of GA in response to osmotic stress in *Arabidopsis* seedlings [78], confirming the role of plant hormones in different physiochemical responses during oxidative stress caused by abiotic stress [79]. Moreover, our results show accumulated IAA-Asp and IAA-Glu during seed germination under salt stress. In *Pisum sativum*, IAA-Asp has a protective role under salt and cadmium stress, demonstrating its relation with plant response to environmental stresses, which possibly indicates the signaling function, and IAA-Asp has a direct biological function in abiotic stresses [80].

### 4.3. Metabolic Pathway Analysis 

**Figure 8:**
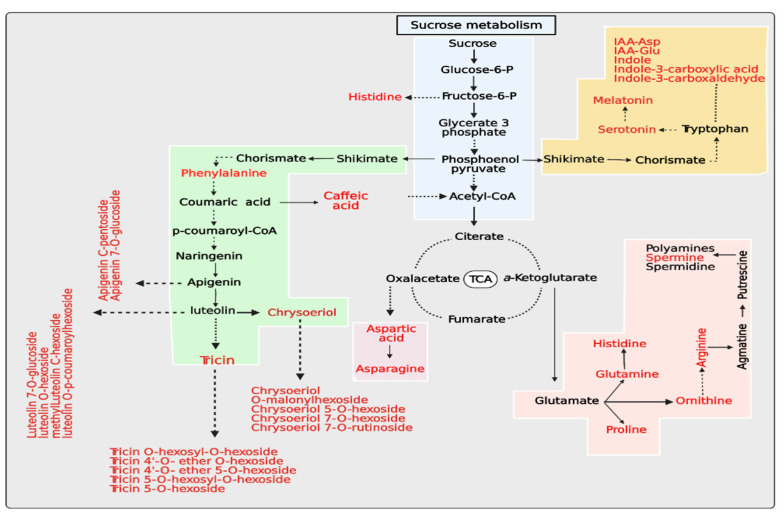
Proposed schematic of top differential metabolites of Yangyou 9 (T) and Zhengsheng 11 (S) exposed to salinity conditions.

Salt stress induces molecular reprogramming, which plays a significant role in rapeseed seeds under stress. Reduced water availability and/or ionic imbalance due to salinity stress might influence different pathways. A wide range of metabolite compounds are accumulated in response to salinity stress from a functional viewpoint; additionally, amino acids, sucrose, and flavonoids are the most common metabolites that accumulate in response to salinity stress in two cultivars (Yangyou 9 and Zhongshuang 11), as shown in Figure 8.

## 5. Conclusions

Among the studied rapeseed cultivars, Yangyou 9 showed higher growth, while Zhongshuang 11 displayed a remarkably low growth rate under stress induced by 150 and 200 mM of NaCl. Higher osmolyte accumulation in Yangyou 9 reduced salt-induced oxidative stress by direct or indirect ROS degeneration and induced antioxidant enzyme activity. A comprehensive examination of metabolite changes under salt stress showed that, out of 332 compounds detected in the metabolic profile, 225 metabolites were filtrated. While the concentrations of inosine, spermine, vitamin B2, tricin O-hexosyl-O-hexoside, D-(+)-maltose, IAA-Asp, and indole were increased within both cultivars, several key metabolites—cholesterol, L-aspartic acid, L-asparagine, ornithine, N-feruloyl serotonin, pyridoxine, linolenic acid, inosine 5’-monophosphate and phosphoric acid were accumulated in Yangyou 9 only. Amino acid, sucrose, and flavonoids are the most common metabolites that can accumulate due to salt stress. Our study provides a sound basis for examining the salt tolerance of various rapeseed cultivars, and Yangyou 9 exhibited higher tolerance against salinity stress, which might be a significant germplasm resource for breeding programs that aim to develop salt-tolerant rapeseed.

Moreover, the identified metabolites can act as biomarkers to characterize plant performance under salt stress in breeding programs (as one promising strategy). Finally, the present study provides a framework for fundamental biochemical analyses and an informed database that can be used to recognize salt-tolerant and -sensitive characteristics before developing a salinity tolerance breeding program for rapeseed cultivars. Accordingly, our findings could be used for further phenotypic and genotypic association studies. This knowledge is of great value for plant breeders aiming at developing new rapeseed cultivars under stress conditions.

## Figures and Tables

**Table 1:** Na^+^, K^+^, and Na^+^/K^+^ ratio in shoots under salt stress during the early seedling stage.

Traits	Na^+^ (mg/g)		K^+^ (mg/g)		Na^+^/K^+^ (mg/g)	
Cultivars	Yangyou 9	Zhongshuang 11	Yangyou 9	Zhongshuang 11	Yangyou 9	Zhongshuang 11
**CK**	3.82 ± 0.21 ^c^	5.09 ± 0.25 ^c^	7.88 ± 0.30 ^a^	6.77 ± 0.21 ^ab^	0.48 ± 0.05 ^c^	0.75 ± 0.06 ^c^
**NaCl**	30.47 ± 0.55 ^b^	48.76 ± 0.61 ^a^	6.83 ± 0.41 ^a^	4.87 ± 0.34 ^b^	4.46 ± 0.02 ^b^	10.01 ± 0.07 ^a^

Data are the mean (± SE) of three replicates. Letters (a, b and c) on vertical bars represent significant differences between cultivars and treatments according to Duncan’s multiple range test (DMRT) at *p* < 0.05.

## Data Availability

Data is contained within the article.

## Supplementary Materials

The following are available online at https://www.mdpi.com/article/10.3390/antiox10081227/s1, Supplementary file (1), Figure S1: Impact of NaCl treatments on (a) shoot length (cm), (b) root length (cm), (c) shoot fresh weight (g), (d) root fresh weight (g), I shoot dry weight (g), and (f) root dry weight (g) of five rapeseed cultivars during early seedling stage, Figure S2: Impact of NaCl treatments on (a) seedling fresh weight stress index, and (b) seedling length stress index of five rapeseed cultivars during the germination stag, Figure S3: Correlation among different growth and biochemical attributes of rapeseed cultivars, Figure S4: Up- and down-regulation of differential metabolites of Yangyou 9 (T) and Zhengsheng 11 (S). (a) Heat-map; (b) Correlation; (c) Scoring plot and (d) Loading plot of principal component analysis (PCA) of differential metabolites during 12, 24h of germination upon salt treatment for tolerant cultivar (T) and sensitive cultivar (S), Table S1: Means of FG%, GR, VI (I), and VI (II) of the studied cultivars in the germination stage under salinity stress during the seed germination stage, Table S2: Metabolites differentially accumulated in Yangyou 9 and Zhongshuang 11 seeds under salt stress (150 mM NaCl). Supplementary file (2), Identified metabolites list.

## Abbreviations

AAs	Amino acid
APX	Ascorbate peroxidase
CAT	Catalase
Chl	Chlorophyll
DAB	3,3-diaminobenzidine
FC	Fold change
FW	Fresh weight
MDA	Malondialdehyde
NBT	Nitro blue tetrazolium
POD	Peroxidase
PA	Polyamine
SOD	Superoxide dismutase
TCA	Tricarboxylic acid
TSS	Total soluble sugar
TSP	Total soluble protein
