# Supplementary material for: Antioxidative and Metabolic Contribution to Salinity Stress Responses in Two Rapeseed Cultivars during the Early Seedling Stage
Source: Antioxidants (Basel). 2021 Jul 30;10(8):1227. doi: 10.3390/antiox10081227 (PMC8389040; doi:10.3390/antiox10081227)
Supplement: Supplementary file 1 [file antioxidants-10-01227-s001.zip › antioxidants-1270326-supplementary.pdf]

Table S1. Means of FG%, GR, VI (I), and VI (II) of the studied cultivars in the germination stage under salinity stress during the seed germination stage.

Treatments	Germination traits	Yangza 11	Zhongshuang 11	Huayouza 62	Fengyou 520	Yangyou 9
0 mM	FG%	97.00±0.6^{a-e}	92.00±1.2^{g}	97.83±0.6^{a-d}	99.50±0.3^{a}	95.83±1.2^{b-f}
	GR	96.42±0.5^{bc}	77.09±0.5^{j}	95.01±0.2^{cd}	98.38±0.4^{a}	94.69±0.5^{d}
	VI (I)	725.6±7.5^{cd}	715.7±7.5^{de}	812.8±2.4^{b}	1011±6.0^{a}	761.1±9.6^{c}
	VI (II)	45.64±0.5^{gh}	39.25±0.9^{i}	54.62±0.8^{d}	53.32±0.9^{de}	50.11±0.7^{ef}
50 mM	FG%	99.67±0.3^{a}	86.00±1.2^{h}	96.83±0.6^{a-e}	99.67±0.3^{a}	98.00±0.6^{a-d}
	GR	91.09±0.7^{f}	61.81±0.7^{m}	92.82±0.4^{e}	97.71±0.7^{ab}	94.72±0.4^{d}
	VI (I)	674.3±4.5^{e}	559.1±9.0^{f}	699.6±3.5^{de}	814.6±2.7^{b}	1039±5.6^{a}
	VI (II)	55.37±0.8^{d}	49.46±1.4^{f}	73.69±1.4^{a}	69.71±1.9^{b}	73.60±0.5^{a}
100 mM	FG%	98.33±0.9^{abc}	71.83±1.2^{i}	94.33±0.9^{efg}	99.00±0.6^{ab}	97.83±1.2^{a-d}
	GR	69.56±0.8^{k}	50.45±0.4^{n}	83.36±0.4^{h}	96.73±0.2^{b}	93.82±0.1^{de}
	VI (I)	442.2±0.7^{h}	274.8±1.1^{i}	511.3±0.8^{g}	564.5±7.9^{f}	828.4±9.0^{b}
	VI (II)	43.65±0.4^{h}	33.70±0.1^{j}	62.22±1.3^{c}	63.78±2.4^{c}	63.77±2.6^{c}
150 mM	FG%	95.17±1.5^{c-f}	50.83±0.6^{k}	86.83±1.7^{h}	94.83±1.2^{defg}	96.00±1.2^{b-f}
	GR	65.94±0.6^{l}	32.63±0.3^{r}	81.16±0.5^{i}	75.68±0.4^{g}	93.97±0.4^{de}
	VI (I)	276.9±7.5^{i}	136.1±3.2^{k}	312.9±9.2^{i}	315.6±4.2^{i}	441.5±1.7^{h}
	VI (II)	33.59±0.2^{j}	14.69±0.4^{m}	37.78±1.0^{i}	37.66±0.4^{i}	47.95±1.5^{fg}
200 mM	FG%	83.83±1.2^{h}	24.00±1.2^{m}	45.83±1.2^{l}	62.50±1.4^{j}	92.83±1.2^{fg}
	GR	43.43±0.6^{o}	12.68±0.4^{s}	37.62±0.4^{q}	40.02±0.5^{p}	87.77±0.4^{g}
	VI (I)	209.0±1.1^{j}	44.65±1.7^{m}	91.77±3.9^{l}	75.78±0.3^{lm}	204.0±3.9^{j}
	VI (II)	23.27±0.7^{l}	5.02±0.3^{n}	14.06±0.3^{m}	15.40±0.5^{m}	30.35±0.5^{k}

FG%: final germination percentage; GR: germination rate; VI (I): vigor index I, and VI (II): vigor index II. Data presented are mean (±SE) of three replicates. The difference in letters indicates significant differences at ($P < 0.05$) using Duncan's multiple range tests.

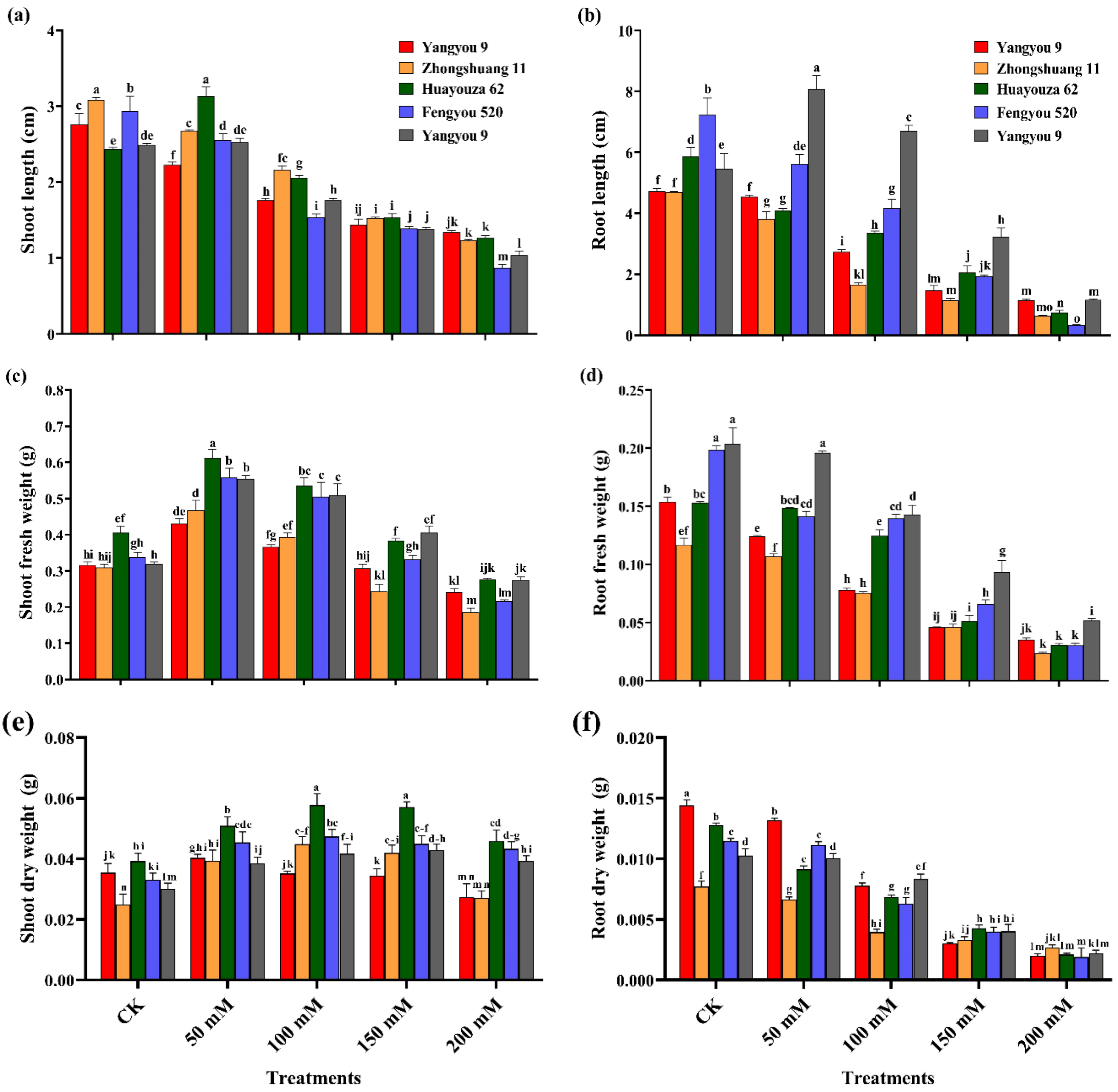

Figure S1. The impact of NaCl treatments on (a) shoot length (cm); (b) root length (cm); (c) shoot fresh weight (g); (d) root fresh weight (g); (e) shoot dry weight (g), and (f) root dry weight (g) of five rapeseed cultivars during the early seedling stage. Bars represent ± SE of three replicates. The difference in letters indicates significant differences at ($P < 0.05$) using Duncan's multiple range tests.

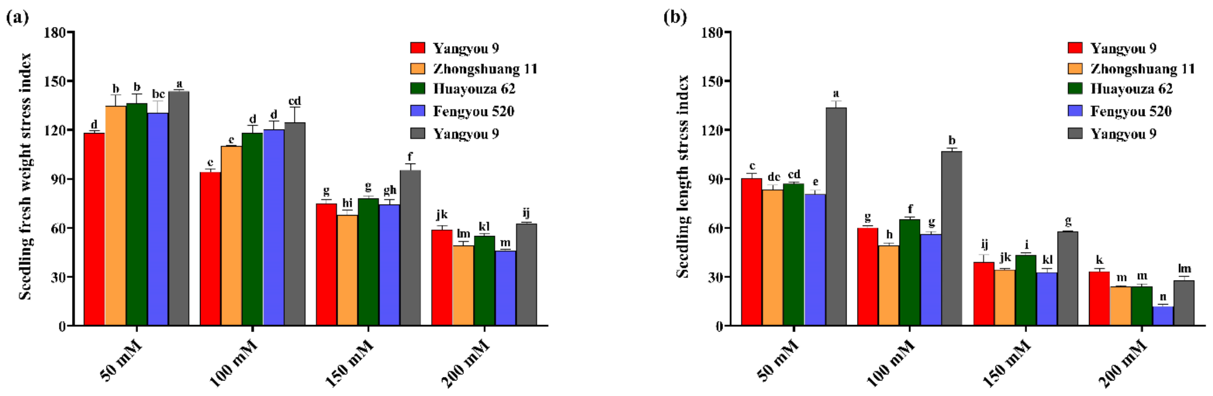

Figure S2. The impact of NaCl treatments on (a**)** seedling fresh weight stress index and (b) seedling length stress index of five rapeseed cultivars during the germination stage. Bars represent ± SE of three replicates. The difference in letters indicates significant differences at (P < 0.05) using Duncan's multiple range tests.

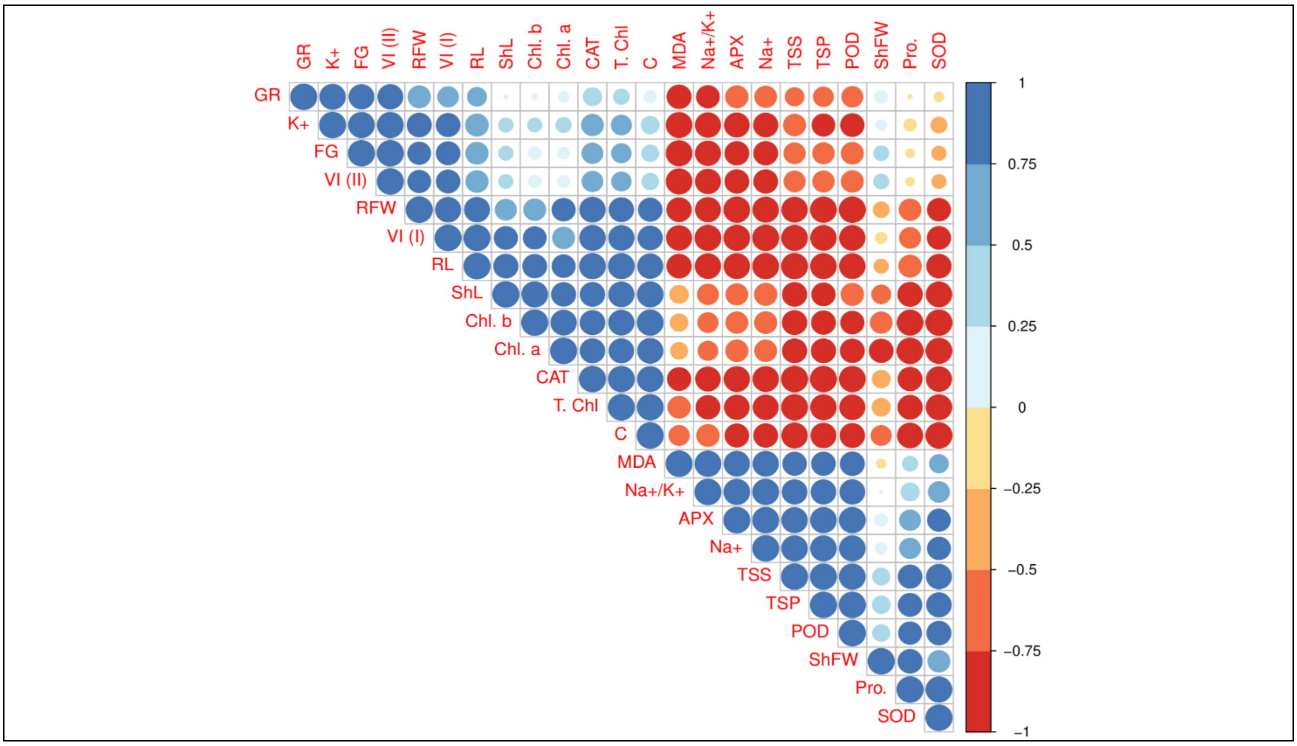

Figure S3. Correlation among different growth and biochemical attributes of rapeseed cultivars. FG: final germination %; GR: germination rate; VI (I): vigor index I; VI (II): vigor index II; SL: shoot length; RL: root length; ShFW: shoot fresh weight; RFW: fresh root weight; Chl. a: chlorophyll a; Chl. b: chlorophyll b; T. Chl: total chlorophyll; C: carotenoid content; TSS: total soluble sugar; TSP: total soluble protein; pro.: proline %; MDA: malondialdehyde content; Na^+: sodium ions; K^+: potassium ions; SOD: superoxidase activity; POD: peroxidase activity; APX: ascorbate peroxidase activity, and CAT: catalase activity.

Table S2. Metabolites differentially accumulated in Yangyou 9 and Zhongshuang 11 seeds upon salt stress (150 mM NaCl).

Metabolites	Tolerant cultivar NaCl.vs.CK (12 h)		Sensitive cultivar NaCl.vs.CK (12 h)		Tolerant cultivar NaCl.vs.CK (24 h)		Sensitive cultivar NaCl.vs.CK (24 h)	
	FC	P-value	FC	P-value	FC	P-value	FC	P-value
**Fatty acid**								
MAG (18:3)	1.47	0.000	1.05	0.029	1.07	0.001	1.07	0.000
MAG (18:2)	1.07	0.000	0.82	0.000	0.76	0.000	0.87	0.000
MGMG (18:2)	0.49	0.000	0.49	0.000	0.42	0.000	0.50	0.000
DGMG (18:1)	0.80	0.000	0.90	0.000	0.66	0.000	0.80	0.000
DGMG (18:2)	0.90	0.000	0.92	0.002	0.86	0.000	0.86	0.001
LPE (18:2)	0.91	0.000	0.84	0.000	1.04	0.011	0.92	0.001
LysoPE 18:2	0.73	0.000	0.96	0.020	0.59	0.000	1.52	0.000
α-Linolenic acid	0.89	0.000	0.73	0.000	0.94	0.000	0.75	0.000
Punicic acid	0.89	0.001	0.85	0.000	0.96	0.004	0.80	0.000
Tianshic acid	0.94	0.008	1.15	0.000	0.87	0.002	0.68	0.000
Cholesterol	1.12	0.000	0.91	0.000	0.82	0.000	0.90	0.001
Linolenic acid	0.89	0.000	0.93	0.000	1.22	0.000	0.85	0.002
14,15-Dehydrocrepenynic acid	0.94	0.014	1.10	0.000	1.09	0.000	0.76	0.000
sn-Glycero-3-phosphocholine	0.78	0.000	0.78	0.000	0.79	0.000	0.77	0.000
**Amino acid**								
L-Alanine	0.80	0.000	0.68	0.000	0.76	0.002	0.77	0.000
L-Serine	0.92	0.001	0.89	0.002	0.78	0.012	0.77	0.000
L-Valine	0.90	0.003	0.80	0.003	0.83	0.000	0.95	0.009
L-Threonine	0.85	0.000	0.88	0.011	0.74	0.000	0.80	0.001
L-Leucine	0.91	0.004	0.86	0.001	0.79	0.000	0.92	0.012
L-Isoleucine	0.90	0.002	0.90	0.003	0.80	0.000	0.92	0.005
L-Aspartic acid	1.17	0.010	0.93	0.049	0.80	0.010	0.91	0.003
L-Lysine	0.93	0.011	0.94	0.001	0.76	0.000	0.85	0.002
L-Glutamic acid	0.88	0.006	0.84	0.004	0.91	0.007	0.85	0.000
L-Methionine	0.91	0.032	0.82	0.003	0.87	0.000	0.81	0.000
L-Histidine	1.43	0.000	1.37	0.000	1.08	0.000	1.06	0.012
L-Phenylalanine	0.91	0.005	0.85	0.001	0.94	0.009	0.88	0.003
L-Arginine	1.74	0.000	1.13	0.001	1.38	0.001	1.15	0.000
L-Tyrosine	0.87	0.001	0.79	0.012	0.78	0.003	0.92	0.008
L-Tryptophan	0.93	0.012	0.88	0.001	0.69	0.002	0.85	0.014
L-Tryptamine	0.94	0.002	1.05	0.001	0.75	0.005	0.92	0.002
L-Asparagine	1.11	0.001	0.87	0.001	0.90	0.002	0.92	0.004
L-Tyramine	0.90	0.013	1.04	0.002	0.82	0.001	1.13	0.000
L-Proline	1.11	0.000	1.07	0.000	1.11	0.000	1.17	0.000
L-Methionine sulfone	0.88	0.000	1.00	0.033	0.79	0.000	0.96	0.000
Ornithine	1.10	0.000	0.97	0.038	0.89	0.000	0.91	0.001
beta-Homothreonine	1.12	0.006	0.89	0.016	0.70	0.000	0.86	0.000
Cysteine	0.72	0.000	1.14	0.007	0.91	0.000	0.66	0.000
5-Hydroxytryptophan	1.04	0.025	0.91	0.000	0.85	0.000	0.93	0.000
Glutathione (oxidized form)	1.17	0.000	0.76	0.000	1.09	0.000	0.81	0.000
Saccharopine	0.99	0.713	0.97	0.473	1.09	0.017	1.40	0.001
Leucine derivative	1.17	0.007	1.20	0.000	1.07	0.000	1.28	0.000
Kynurenine	1.13	0.004	1.33	0.001	1.29	0.000	1.43	0.000
N-p-Coumaroyltryptamine	1.12	0.010	1.32	0.001	1.09	0.014	1.21	0.000
3,4-Dihydroxy-L-phenylalanine	0.80	0.000	0.87	0.263	1.42	0.000	1.10	0.000
4-Guanidinobutanoate	0.96	0.000	1.04	0.000	0.88	0.000	0.92	0.000
Xanthurenic acid	0.94	0.179	1.15	0.000	1.07	0.000	0.77	0.000
Kynurenic acid	1.02	0.000	1.06	0.001	0.88	0.000	0.90	0.000
Serotonin	0.69	0.000	1.15	0.000	0.83	0.000	1.13	0.000
Xanthurenic acid O-hexoside	1.02	0.000	0.92	0.000	0.90	0.000	1.02	0.000
N-Benzoylserotonin	1.01	0.001	0.94	0.000	0.80	0.000	1.14	0.000
N-p-Coumaroylserotonin	1.12	0.000	0.93	0.000	0.76	0.000	0.94	0.000
N-Feruloylserotonin	1.11	0.000	0.91	0.000	0.99	0.005	0.99	0.010
N-Acetyl-L-leucine	1.02	0.000	1.03	0.000	0.98	0.000	0.98	0.000
N-Acetyltryptamine	1.12	0.000	1.14	0.000	1.05	0.005	0.78	0.000

(continued on next page)

Table S2. (continued)

Metabolites	Tolerant cultivar NaCl.vs.CK (12 h)		Sensitive cultivar NaCl.vs.CK (12 h)		Tolerant cultivar NaCl.vs.CK (24 h)		Sensitive cultivar NaCl.vs.CK (24 h)	
	FC	P-value	FC	P-value	FC	P-value	FC	P-value
**Nucleic acid**								
Guanine	0.87	0.000	0.84	0.001	0.92	0.001	0.84	0.000
Xanthine	0.81	0.000	0.67	0.000	0.95	0.029	0.79	0.000
Uridine	1.34	0.001	1.26	0.002	1.49	0.000	1.37	0.011
Inosine	1.08	0.002	1.10	0.002	1.30	0.000	1.14	0.030
Xanthosine	0.94	0.029	0.88	0.042	1.56	0.000	0.86	0.005
Uracil	1.24	0.001	1.11	0.004	0.84	0.001	1.34	0.000
Adenine	0.92	0.001	0.80	0.000	0.93	0.006	0.71	0.000
Cytidine	1.08	0.016	1.63	0.000	1.08	0.030	1.08	0.003
Crotonoside	1.05	0.042	0.85	0.000	1.57	0.000	0.87	0.033
Guanosine	1.06	0.010	0.88	0.004	1.88	0.000	0.88	0.012
Adenosine	1.23	0.000	1.23	0.004	1.24	0.000	1.04	0.000
N2, N2-Dimethylguanosine	1.09	0.030	0.94	0.015	1.16	0.017	0.98	0.051
trans-zeatin N-glucoside	1.04	0.042	0.96	0.001	0.96	0.000	0.93	0.001
Inosine 5'-monophosphate	0.84	0.000	0.55	0.000	1.11	0.011	0.69	0.001
Succinyl adenosine	0.98	0.027	0.95	0.038	0.90	0.018	1.18	0.001
Adenosine O-ribose	0.95	0.000	0.97	0.043	0.97	0.004	1.08	0.023
N-(9H-Purin-6-ylcarbamoyl) threonine	1.07	0.002	0.91	0.019	1.04	0.006	1.02	0.055
2'-Deoxyadenosine	1.07	0.004	1.04	0.017	1.04	0.034	0.88	0.003
2'-Deoxyinosine-5'-monophosphate	0.29	0.000	0.22	0.000	0.26	0.000	0.21	0.000
Adenosine 3'-monophosphate	0.86	0.000	0.56	0.000	1.01	0.358	0.57	0.006
5'-Deoxy-5'-(methylthio) adenosine	0.69	0.000	0.79	0.001	0.54	0.000	0.77	0.000
**Polyphenols**								
Quinic acid	1.44	0.000	1.21	0.001	1.31	0.000	1.28	0.000
Sinapic acid	1.48	0.000	1.23	0.001	1.77	0.000	1.26	0.000
Ferulic acid	1.16	0.002	1.06	0.039	1.04	0.015	1.10	0.036
Catechin	1.16	0.005	0.86	0.036	1.06	0.031	0.75	0.001
Chlorogenic acid	1.11	0.003	0.80	0.001	0.97	0.095	1.08	0.004
N-Feruloylserotonin	1.37	0.000	0.78	0.000	1.08	0.000	0.89	0.003
Ferulic acid O-hexoside	1.08	0.002	1.04	0.017	0.94	0.003	0.94	0.025
Coniferyl aldehyde	1.20	0.000	1.07	0.039	0.86	0.003	1.13	0.015
Caffeic acid	0.95	0.005	1.13	0.011	0.87	0.000	0.91	0.003
Sinapoyl quinic acid	0.96	0.087	0.95	0.049	0.97	0.112	0.93	0.022
p-Coumaric acid	0.85	0.006	0.91	0.008	0.87	0.000	0.91	0.016
Benzamidine	0.95	0.025	0.69	0.000	0.78	0.002	1.06	0.000
Caffeoyl shikimic acid	0.95	0.016	0.88	0.012	0.98	0.091	0.95	0.007
4-O-p-Coumaroylquinic acid	0.97	0.246	0.91	0.001	0.78	0.006	0.93	0.031
Sinapoyl O-hexoside	0.84	0.001	0.91	0.002	0.85	0.004	0.92	0.040
Fer quinic acid	0.92	0.000	0.86	0.000	0.93	0.000	0.92	0.000
**Phenol amine**								
N-Caffeoylputrescine	1.13	0.000	1.09	0.000	1.18	0.000	1.14	0.000
N-p-Coumaroylputrescine	1.28	0.001	1.11	0.002	1.15	0.000	1.16	0.000
N', N''-p-Coumaroyl Feruloylspermidine	1.21	0.000	1.12	0.008	1.15	0.002	1.10	0.040
p-Coumaroyl-2-hydroxyputrescine	1.09	0.022	1.10	0.000	0.94	0.005	0.94	0.005
N-p-Coumaroylagmatine	0.74	0.000	0.83	0.000	0.85	0.000	0.91	0.029
N-Feruloylagmatine	0.83	0.014	0.65	0.000	0.79	0.000	0.63	0.000
N', N''-Di-p-Coumaroylspermidine	0.87	0.001	1.14	0.000	0.81	0.000	1.09	0.000
N-p-Coumaroylputrescine derivative	0.88	0.001	0.91	0.003	0.79	0.000	0.77	0.000
N-Feruloyl Cadaverine	0.88	0.000	0.83	0.000	0.78	0.000	0.72	0.000
**Polyamine**								
Spermine	1.20	0.000	1.55	0.000	1.23	0.001	1.25	0.000
Feruloyl putrescine O-hexoside	0.81	0.002	0.90	0.005	0.72	0.000	0.86	0.001
**Alkaloid**								
Trigonelline	1.26	0.000	1.05	0.036	1.15	0.002	0.89	0.013
Betaine	1.10	0.000	0.85	0.044	1.05	0.002	0.90	0.011

(continued on next page)

Table S2. (continued)

Metabolites	Tolerant cultivar NaCl.vs.CK (12 h)		Sensitive cultivar NaCl.vs.CK (12 h)		Tolerant cultivar NaCl.vs.CK (24 h)		Sensitive cultivar NaCl.vs.CK (24 h)	
	FC	P-value	FC	P-value	FC	P-value	FC	P-value
**Vitamins**								
Vitamin B2	1.07	0.005	1.23	0.000	1.20	0.000	1.06	0.049
Pyridoxine O-glucoside	1.28	0.002	1.08	0.000	1.18	0.002	0.80	0.002
Thiamin	1.21	0.000	1.04	0.008	1.09	0.001	1.07	0.000
4-Pyridoxic acid O-hexoside	1.18	0.002	0.86	0.000	0.91	0.016	1.25	0.001
4-Methyl-5-thiazoleethanol	1.26	0.000	1.02	0.051	1.23	0.000	1.43	0.000
Carbachol	1.05	0.001	1.04	0.003	1.24	0.000	0.79	0.000
Nicotinic acid	1.10	0.000	1.03	0.015	1.06	0.000	1.16	0.002
1-Methylnicotinamide	1.07	0.002	0.88	0.000	1.04	0.000	0.95	0.012
Pyridoxine	1.07	0.009	0.86	0.000	0.90	0.000	0.92	0.000
Niacinamide	0.88	0.026	0.53	0.000	1.15	0.002	0.75	0.000
Nicotinoylcholine	1.09	0.004	0.94	0.054	1.07	0.002	0.89	0.001
Choline	1.23	0.001	1.05	0.000	1.17	0.005	1.48	0.000
Sinapoylcholine	1.10	0.000	0.92	0.000	1.06	0.000	0.94	0.020
**Flavonoid**								
Apigenin C-pentoside	1.22	0.000	1.09	0.002	0.75	0.000	0.92	0.040
Apigenin 6-C-glucoside	0.82	0.002	0.65	0.001	0.90	0.035	0.73	0.001
Apigenin 7-O-glucoside	1.09	0.000	1.24	0.002	1.20	0.000	1.31	0.001
C-pentosyl-apigenin O-hexoside	1.13	0.001	1.18	0.001	1.10	0.001	1.06	0.014
C-hexosyl-luteolin O-hexoside	1.32	0.001	1.23	0.015	1.13	0.031	1.23	0.000
C-hexosyl-luteolin O-p-coumaroylhexoside	1.32	0.000	1.17	0.000	1.25	0.000	1.09	0.004
Chrysoeriol	1.30	0.002	1.10	0.169	1.39	0.000	1.32	0.000
Chrysoeriol C-hexoside	0.96	0.051	0.88	0.040	0.85	0.014	0.79	0.000
Chrysoeriol O-malonylhexoside	1.19	0.001	0.97	0.458	1.11	0.015	0.97	0.403
Chrysoeriol 5-O-hexoside	1.21	0.020	1.06	0.015	0.98	0.036	0.95	0.013
Chrysoeriol 7-O-hexoside	1.08	0.027	1.11	0.000	1.03	0.001	1.03	0.022
Chrysoeriol 7-O-rutinoside	1.05	0.017	1.10	0.000	1.23	0.000	1.08	0.008
Tricin	1.19	0.013	0.70	0.000	1.09	0.001	0.81	0.000
Tricin O-hexosyl-O-hexoside	1.06	0.056	1.09	0.024	1.06	0.048	1.05	0.021
Tricin 4'-O- (syringyl alcohol) ether O-hexoside	1.01	0.765	0.73	0.000	1.18	0.000	1.22	0.000
Tricin 4'-O-(syringyl alcohol) ether 5-O-hexoside	1.17	0.004	1.62	0.000	1.17	0.011	1.50	0.000
Tricin 5-O-hexosyl-O-hexoside	1.15	0.003	1.23	0.005	0.96	0.055	1.01	0.284
Tricin 5-O-hexoside	1.06	0.098	1.02	0.186	1.02	0.687	1.02	0.048
Tricin 7-O-hexoside	0.98	0.048	0.93	0.000	0.88	0.007	0.85	0.002
Phellodensin F	0.91	0.004	0.92	0.022	0.60	0.000	0.76	0.002
Luteolin 6-C-glucoside	0.62	0.001	0.87	0.032	0.56	0.001	0.88	0.032
Luteolin 7-O-glucoside	1.13	0.010	1.20	0.027	0.85	0.041	1.30	0.000
C-pentosyl-apeignin O-feruloylhexoside	0.98	0.366	0.93	0.017	0.92	0.009	0.85	0.000
Glycitin	0.92	0.036	1.41	0.000	0.87	0.022	1.16	0.003
methylLuteolin C-hexoside	1.19	0.000	1.27	0.000	1.06	0.023	1.05	0.025
Selgin 5-O-hexoside	1.10	0.021	0.93	0.000	1.19	0.000	0.92	0.002
Cyanidin 3,5-di-O-hexoside	1.03	0.033	0.94	0.007	1.16	0.053	0.89	0.026
Cyanidin 3-O-glucoside	0.85	0.000	0.93	0.000	0.87	0.000	0.78	0.000
Delphinidin O-rutinoside	1.05	0.054	0.72	0.000	0.74	0.000	0.90	0.000
**Sugars**								
Fructose 1, 6-diphosphate	1.20	0.001	0.74	0.000	1.16	0.006	1.13	0.000
Sucrose	1.05	0.009	0.93	0.002	1.06	0.001	1.05	0.032
D- (+)-Maltose	2.56	0.000	2.58	0.000	2.59	0.000	2.40	0.000
α-Lactose	1.08	0.003	0.90	0.000	1.06	0.001	0.90	0.000
a-L-Rhamnose	0.90	0.011	0.94	0.001	0.92	0.019	0.96	0.002
**Terpene**								
Polygodial	1.33	0.001	0.87	0.003	1.10	0.039	0.77	0.003
Diosgenin	0.91	0.011	1.10	0.020	1.13	0.027	1.12	0.014

(continued on next page)

Table S2. (continued)

Metabolites	Tolerant cultivar NaCl.vs.CK (12 h)		Sensitive cultivar NaCl.vs.CK (12 h)		Tolerant cultivar NaCl.vs.CK (24 h)		Sensitive cultivar NaCl.vs.CK (24 h)	
	FC	P-value	FC	P-value	FC	P-value	FC	P-value
**Hormones**								
Gibberellin A14	1.07	0.003	1.24	0.000	1.20	0.000	0.66	0.000
IAA	0.89	0.000	0.97	0.029	0.96	0.251	1.02	0.048
IAA-Asp	1.46	0.000	1.38	0.000	2.44	0.000	1.72	0.000
IAA-Glu	1.13	0.001	0.91	0.012	1.07	0.004	1.26	0.007
Indole	1.26	0.000	1.16	0.000	1.80	0.000	1.19	0.000
Indole-3-carboxylic acid	0.84	0.000	1.06	0.014	0.91	0.000	1.08	0.001
Indole-3-carboxaldehyde	1.19	0.000	1.22	0.004	1.24	0.004	1.16	0.001
Indoline	0.98	0.006	0.91	0.000	0.84	0.001	0.95	0.012
Melatonin	1.19	0.000	0.92	0.010	1.23	0.000	0.84	0.000
Methoxy indoleacetic acid	0.96	0.018	1.03	0.037	0.98	0.287	1.08	0.004
**Others**								
Tuberonic acid hexoside	0.84	0.003	0.95	0.009	0.89	0.016	0.88	0.001
DIMBOA glucoside	1.11	0.000	0.66	0.000	1.15	0.000	0.91	0.000
Nicotianamine	1.05	0.004	0.89	0.014	1.10	0.001	1.29	0.000
Roseoside	1.06	0.000	1.06	0.032	0.93	0.033	0.93	0.009
Manglieside E	1.06	0.040	0.58	0.000	1.15	0.002	1.11	0.007
O-Acetyl-L-serine	0.92	0.021	0.91	0.028	0.95	0.012	0.95	0.027
DL-alpha, epsilon-Diaminopimelic acid	0.94	0.002	1.03	0.495	0.84	0.000	1.02	0.020
Pipecolinic acid	0.97	0.011	0.94	0.009	0.87	0.005	0.89	0.010
p-Nitroaniline	1.22	0.001	1.34	0.001	1.12	0.001	1.17	0.004
2-(5-hydroxy-1H-indol-3-yl) acetic acid	1.19	0.004	1.13	0.000	0.91	0.012	1.15	0.003
Carnitine	0.82	0.002	0.87	0.018	0.73	0.000	0.82	0.009
N-acetylneuraminic acid	1.18	0.000	0.91	0.008	0.78	0.000	0.83	0.001
Glucosamine	0.78	0.001	0.72	0.000	0.85	0.001	0.74	0.003
1-methyladenosine	0.72	0.000	1.32	0.000	1.91	0.000	1.22	0.000
1-methylguanosine	0.95	0.007	0.88	0.000	0.90	0.006	0.89	0.000
5-methylcytidine	0.80	0.005	0.55	0.000	0.68	0.000	0.45	0.000
2'-O-methyladenosine	1.12	0.055	1.18	0.000	1.43	0.006	1.08	0.013
2'-O-methylguanosine	1.05	0.003	0.93	0.000	1.32	0.000	1.17	0.000
N4-Acetylsulfamethoxazole	0.88	0.000	0.89	0.001	0.89	0.001	0.73	0.000
Pinoresinol 4-O-glucoside	1.27	0.003	0.77	0.000	1.39	0.000	0.74	0.000
Isobornyl methacrylate	1.18	0.001	0.92	0.018	0.96	0.109	0.78	0.000
2-(Carboxyacetamido)benzoic acid	1.35	0.000	1.41	0.000	0.75	0.000	0.72	0.000
1-(beta-D-Ribofuranosyl)-3-pyridiniumcarboxylate	0.94	0.000	1.02	0.012	0.81	0.000	0.89	0.001
Tolycaine	0.88	0.009	1.10	0.001	1.12	0.003	1.32	0.000
Oleamide	0.80	0.000	0.89	0.001	1.13	0.001	0.73	0.000
Gelsemine	0.87	0.000	0.65	0.000	0.58	0.000	0.64	0.000
Golotimod	1.35	0.000	1.17	0.002	1.16	0.000	1.01	0.059
Citroflex 2	0.98	0.020	0.72	0.000	0.97	0.002	0.66	0.000
1-[5-(2,3,4-Trihydroxybutyl)-2-pyrazinyl]-1,2,3,4-butanetetrol	1.17	0.002	0.93	0.005	1.08	0.023	0.91	0.000
2-Aminoisobutyric acid	1.21	0.005	0.86	0.002	0.75	0.000	0.80	0.002
N-Undecanoylglycine	0.95	0.001	0.93	0.004	0.96	0.027	0.95	0.007
(9S,13S)-12-Oxophytodienoic acid	1.23	0.002	1.05	0.000	1.16	0.000	0.84	0.029
Methyl 2-[(2-methoxy-2-oxoethyl) amino] acetate	0.86	0.007	0.90	0.000	0.95	0.024	0.85	0.000
N-acetyl-L-2-aminoadipic acid	1.09	0.000	0.92	0.000	1.16	0.001	0.91	0.001
Urocanic acid	1.05	0.002	1.07	0.002	0.91	0.000	0.93	0.000
DL-3,4-Dihydroxymandelic acid	1.07	0.000	1.68	0.000	1.06	0.008	1.14	0.004
S-Carboxymethyl-L-cysteine	1.28	0.000	1.12	0.001	1.20	0.000	1.15	0.000
4-Pyridoxate	1.18	0.000	1.11	0.006	0.93	0.000	1.14	0.004
Kinetin	1.31	0.000	0.91	0.002	1.06	0.001	1.34	0.001
8-Chlro-1-tetrahydronorharmanone	1.11	0.024	1.17	0.020	1.10	0.003	0.89	0.006
5-Hydroxytryptophan	0.84	0.000	0.80	0.000	0.80	0.000	0.89	0.005
2,3-dihydroflavone	1.98	0.000	1.24	0.001	1.16	0.000	1.12	0.004
7-Hydroxyflavone	1.11	0.000	1.13	0.000	0.91	0.001	0.77	0.000
Caffeoylcholine	0.88	0.038	1.17	0.007	0.84	0.008	1.00	0.843
Sinapoyl malate	1.27	0.001	1.05	0.132	1.09	0.004	0.89	0.000

(continued on next page)

Table S2. (continued)

Metabolites	Tolerant cultivar NaCl.vs.CK (12 h)		Sensitive cultivar NaCl.vs.CK (12 h)		Tolerant cultivar NaCl.vs.CK (24 h)		Sensitive cultivar NaCl.vs.CK (24 h)	
	FC	P-value	FC	P-value	FC	P-value	FC	P-value
Hinokinin	0.91	0.000	0.69	0.000	1.19	0.000	1.26	0.000
3-Methoxy-4-hydroxybenzoic acid O-hexoside	1.33	0.000	1.12	0.007	1.17	0.015	1.13	0.006
4-Indolecarbaldehyde	1.17	0.000	1.22	0.000	1.63	0.000	1.40	0.000
Phosphoric acid	0.92	0.011	0.91	0.014	1.35	0.000	0.64	0.000
Tributyl phosphate	1.19	0.003	1.32	0.000	1.45	0.000	1.19	0.014
ANETHOLE	1.14	0.048	0.90	0.039	1.31	0.000	1.08	0.094
4-hydroxybenzoic acid O-hexoside	1.17	0.001	0.88	0.004	1.10	0.002	0.93	0.001
Inosine	0.94	0.000	1.34	0.000	1.19	0.000	1.13	0.000
Etamiphylline	0.89	0.000	0.90	0.000	1.45	0.000	0.82	0.001
Gingerol	0.79	0.000	1.02	0.291	1.08	0.000	0.91	0.000
Guanosine 5'-monophosphate	1.03	0.002	0.69	0.000	1.50	0.000	0.82	0.000
4-(Heptyloxy)phenol	0.93	0.002	1.05	0.000	1.21	0.000	1.06	0.000
Diethylpyrocarbonate	1.07	0.001	0.91	0.003	0.99	0.360	0.91	0.001
3-[(1R,2S,5R,6S)-5-Hydroxy-7-oxabicyclo [4.1.0] hept-2-yl]-L-alanine	1.14	0.000	1.02	0.054	0.93	0.000	1.18	0.022
Valylserine	0.93	0.158	0.97	0.007	0.83	0.000	1.06	0.001
Cyclo (leucylprolyl)	1.02	0.139	1.12	0.000	0.87	0.000	0.97	0.013
Ketotifen	1.15	0.000	1.03	0.003	1.07	0.001	0.89	0.000
